# Orderly Replication and Segregation of the Four Replicons of *Burkholderia cenocepacia* J2315

**DOI:** 10.1371/journal.pgen.1006172

**Published:** 2016-07-18

**Authors:** Wen-Li Du, Nelly Dubarry, Fanny M. Passot, Alain Kamgoué, Heath Murray, David Lane, Franck Pasta

**Affiliations:** 1 Laboratoire de Microbiologie et Génétique Moléculaires, Centre National de Recherche Scientifique / Université Paul Sabatier, Toulouse, France; 2 Laboratoire de Biologie Moléculaire Eucaryote, Centre National de Recherche Scientifique / Université Paul Sabatier, Toulouse, France; 3 Centre for Bacterial Cell Biology, Institute for Cell & Molecular Biosciences, Newcastle University, Newcastle upon Tyne, United Kingdom; University of Geneva Medical School, SWITZERLAND

## Abstract

Bacterial genomes typically consist of a single chromosome and, optionally, one or more plasmids. But whole-genome sequencing reveals about ten per-cent of them to be multipartite, with additional replicons which by size and indispensability are considered secondary chromosomes. This raises the questions of how their replication and partition is managed without compromising genome stability and of how such genomes arose. *Vibrio cholerae*, with a 1 Mb replicon in addition to its 3 Mb chromosome, is the only species for which maintenance of a multipartite genome has been investigated. In this study we have explored the more complex genome of *Burkholderia cenocepacia* (strain J2315). It comprises an extra replicon (c2) of 3.21 Mb, comparable in size to the3.87Mb main chromosome (c1), another extra replicon(c3) of 0.87 Mb and a plasmid of 0.09 Mb. The replication origin of c1 is typically chromosomal and those of c2 and c3 are plasmid-like; all are replicated bidirectionally. Fluorescence microscopy of tagged origins indicates that all initiate replication at mid-cell and segregate towards the cell quarter positions sequentially, c1-c2-p1/c3. c2 segregation is as well-phased with the cell cycle as c1, implying that this plasmid-like origin has become subject to regulation not typical of plasmids; in contrast, c3 segregates more randomly through the cycle. Disruption of individual Par systems by deletion of *parAB* or by addition of *parS* sites showed each Par system to govern the positioning of its own replicon only. Inactivation of c1, c2 and c3 Par systems not only reduced growth rate, generated anucleate cells and compromised viability but influenced processes beyond replicon partition, notably regulation of replication, chromosome condensation and cell size determination. In particular, the absence of the c1 ParA protein altered replication of all three chromosomes, suggesting that the partition system of the main chromosome is a major participant in the choreography of the cell cycle.

## Author Summary

Unlike higher organisms, bacteria typically carry their genetic information on a single chromosome. But in a few bacterial families the genome includes one to three additional chromosome-like DNA molecules. Because these families are rich in pathogenic and environmentally versatile species, it is important to understand how their split genomes evolved and how their maintenance is managed without confusion. We find that mitotic segregation (partition) of all three chromosomes of the cystic fibrosis type strain, *Burkholderia cenocepacia* J2315, proceeds from mid-cell to cell quarter positions, but that it occurs in a sequential manner, from largest chromosome to smallest. Positioning of each chromosome is specified solely by its own partition proteins. Nevertheless, the partition system of the largest chromosome appears also to play a global role in the cell cycle, by modulating the timing of initiation of replication. In addition, disrupting the partition systems of all three chromosomes induced specific cell abnormalities. Hence, although such bacteria are governed mainly by the largest, housekeeping chromosome, all the Par systems have insinuated themselves into cell cycle regulation to become indispensable for normal growth. Exploration of the underlying mechanisms should allow us to understand their full importance to bacterial life.

## Introduction

The long-held view that bacteria carry the essential part of their genomes on a single chromosome blurred about 25 years ago, when the species *Rhodobacter sphaeroides* was found to carry certain essential genes on a large replicon distinct from the main chromosome [1]. The size and essentiality of this replicon qualified it as a chromosome, albeit a secondary one. Many bacterial genomes have since proven to be multipartite—about 10% of those sequenced and notably those of pathogenic and metabolically versatile species. For example, all Vibrio species carry one secondary chromosome [2,3] and all Burkholderia species have at least one and typically two [4]. They are thought to have arisen by transfer of essential genes to coresident low-copy number plasmids, which thereupon grew through further recombination events. Whether the split-genome arrangements resulting from such events persisted by conferring selective advantage is speculative, but it is reasonable to view expansion of secondary chromosomes as a means of incorporating large numbers of beneficial genes without unduly disturbing the regulation and organization of essential genes on the main chromosome. Our aim here is to determine how the maintenance of one principal and two secondary chromosomes is accommodated within the cell cycle of the beta-proteobacterium *Burkholderia cenocepacia* J2315, an opportunistic pathogen of sufferers from cystic fibrosis. (We use the term "secondary chromosome" for convenience, and deal with the nomenclature of such replicons in the Discussion.)

The size of secondary chromosomes, which can approach that of the main chromosome, makes them potentially problematic. First, the replication control systems of secondary chromosomes resemble those of low-copy number plasmids. Replication of such plasmids has been seen to lack the close coupling with the cell cycle shown by the main chromosome [5–8]; because it occupies only a brief period it is regulated only to ensure that it precedes cell division [9]. Enlargement of such a replicon requires its replication to occupy a large fraction of the cell cycle and so to be initiated early enough not to delay cell division or to risk DNA cleavage by the closing septum. Thus for large secondary chromosomes to be stable, either the system that regulates initiation of plasmid replication must be augmented in some way, or such chromosomes must develop only from plasmids, yet to be identified, that naturally replicate early in the cycle.

Second, the large size of secondary chromosomes risks confusion or entanglement with the main chromosome during the mitotic segregation (partition) that follows their replication. Low-copy number plasmids and most chromosomes assure efficient partition through assembly of a partition complex based on binding of a specific ParB protein to a cluster of *parS* sites near the replication origin; poleward movement of the complex on each replicon copy is then mediated by the cognate ParA ATPase. ParABS systems provide the specificity needed to distinguish between coresident chromosomes [10] but whether they alone can assure orderly movement of bulky chromosome copies is unproven. In this they might be aided through regulatory linkage with replication control. Although replication and Par-mediated segregation operate independently in plasmids, recent work has shown the *Bacillus subtilis* and *Vibrio cholerae* partition proteins to regulate replication-initiator activity as well as chromosome segregation [11–13].

Of the many split-genome cell cycles that might be studied, only that of *V*. *cholerae* has been in depth. The Vc genome comprises chromosomes of 2.9Mb (Chr1) and 1Mb (Chr2). Although the Chr2 origin is plasmid-like, it is replicated in phase with the cycle, but later than Chr1, such that Chr2 and Chr1 replication terminate at about the same time [14]. The question of how the cell manages partition of two bulky replicons has been settled in the case of *V*.*cholerae* by adoption of distinct segregation patterns. The Chr1 origin appears to be tethered at one end of the cell so that segregation consists of moving one origin copy to the far pole where it in turn is fixed, as seen also in *Caulobacter crescentus*, *Agrobacterium tumefaciens*, and *Sinorhizobium meliloti* [15,16], while the Chr2 origin is centrally located in new-born cells whence its copies segregate to the quarter positions prior to division [17], as typified by low copy number plasmids and the single chromosomes of bacterial models such as *B*. *subtilis* and *Pseudomonas aeruginosa* [18,19].

Genomes of the Burkholderia group are divided still further. That of the *B*. *cenocepacia* J2315 reference strain comprises the principal chromosome (c1, 3.9 Mb), two secondary chromosomes (c2, 3.2 Mb, and c3, 0.9 Mb) and a plasmid (p1, 0.09 Mb) [20]. How are these replicons replicated and segregated without confusion and without perturbing the cell cycle? Each carries a *parABS* system which displays non-overlapping specificity in stabilization of plasmids in *E*. *coli* [10] and in formation of partition complexes *in vitro* [21]. Nevertheless, the role of the Par systems in their mother organism has not been defined, nor is it known whether they are brought into play independently or in concert via a cell cycle master regulator. We have examined these issues by characterizing the replication mode of each chromosome, by analyzing the number and positioning of each replicon's *ori-par* region with respect to the cell cycle, and by assessing the consequences for partition and growth of inactivating each ParABS system.

## Results

### *rep-par* regions and replication mode

**Fig 1:**
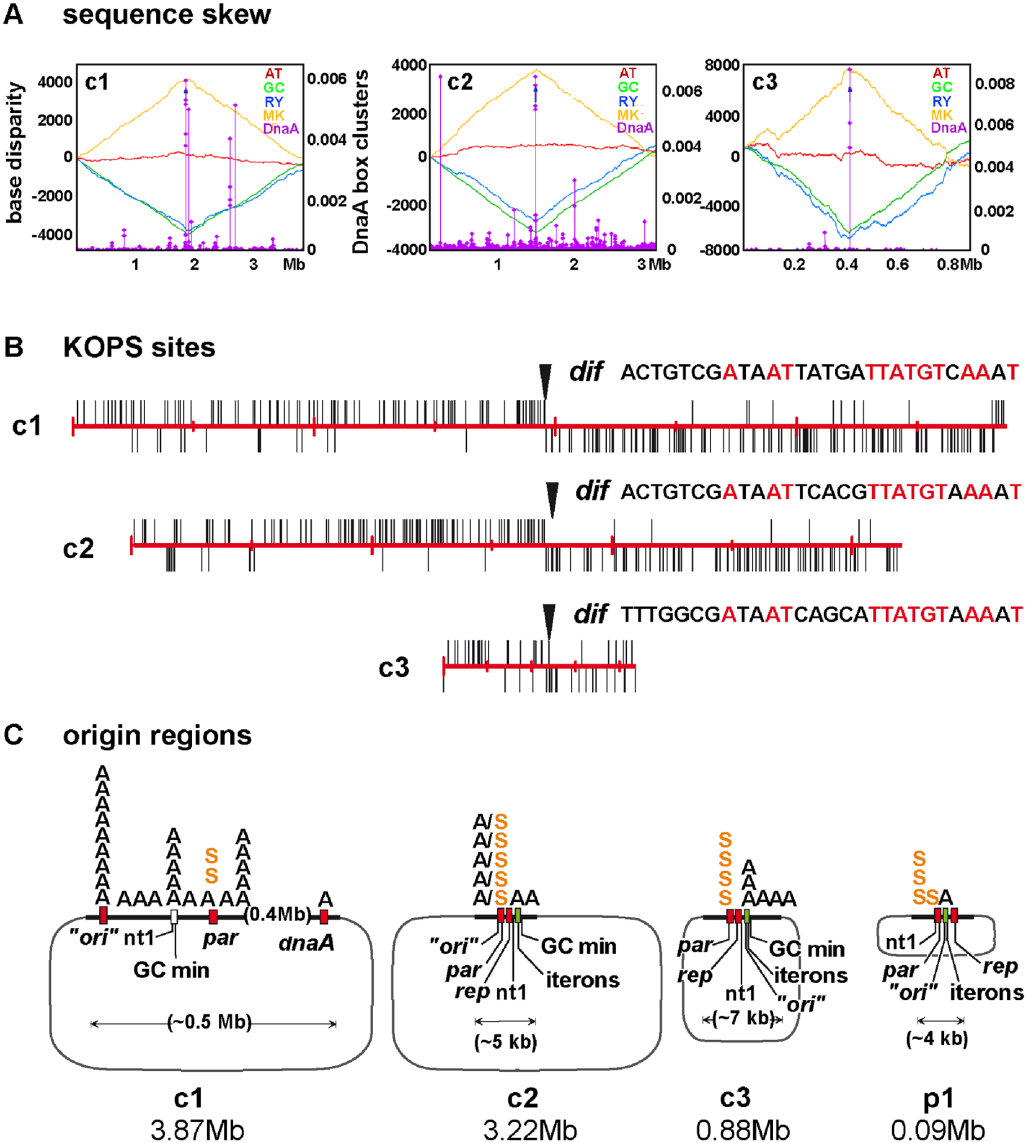
Location of replication regions on the *Bcen* replicons. **A**. Base disparity along c1, c2 and c3, shown as Z-curves (Ori-finder; [22]). Each chromosome sequence is arrayed to place nucleotide 1 at the centre of the x-axis. Z-curves display base distribution asymmetry (left ordinate): with x_n_ = purine vs. pyrimidine (blue trace) and y_n_ = amino vs. keto (yellow trace), GC- disparity (green trace) and AT-disparity (red trace) are defined as (x_n_—y_n_)/2; see [22] for a full explanation. The density of DnaA boxes (right ordinate) is calculated as follows: the distance from each DnaA box (≤ 1 deviation from TTATCCACA) to its adjacent bases are summed and the reciprocals (b values) plotted [24]; each diamond shows a cluster, defined as ≥ 3 DnaA boxes within 100 bp. **B**. Distribution of putative KOP sites on the *Bcen* chromosomes, opened at nucleotide 1 and read clockwise. The sites are assumed to correspond to the *E*.*coli* KOP sequence GGG(C/A/T)AGGG on the basis of nearly complete sharing of conserved and contact residues with the KOPS interaction domain of *E*.*coli* FtsK (S3 Fig; [25]). Arrowheads show *dif* sites corresponding to the Burkholderiales family consensus [26]. **C**. *Ori-par* regions of each chromosome, showing the principal elements (detailed maps are given in S1 and S2 Figs) denoted as follows: “*ori*”—sequence deduced to contain the replication origin by Ori-finder; nt1—nucleotide 1 in the genome database; par–*parAB* partition genes; *rep*–homologue of genes of the replication control protein (RepA) family; iterons—cluster (green rectangles) of 19-21bp sequence repeats characteristic of plasmid replication control elements (detailed in S1 Table); GC min—minimum in GC disparity curve; A—DnaA boxes (those with >1 deviation from the *E*.*coli* TTATCCACA consensus are omitted); S–*parS* sites of ParB binding; A/S–*parS* sites denoted as DnaA boxes by Ori-finder.

To analyze replication of the genome we first characterized the origin regions and determined base-pair frequency gradients throughout each chromosome. The replication origins had been provisionally located from the similarity of their genetic context to that of known origins and from GC-skew minima [10], and we extended this analysis to substantiate the prior indications. Application of the programme Ori-finder 1.0 (Tubic; [22]), designed to identify origins on the basis of DnaA-box density and sequence disparity, confirmed these general locations, as depicted in Fig 1A. The skewed distributions of KOPS sites (Fig 1B), which bind FtsK to facilitate terminus segregation [23], are largely consistent with these *ori* positions. Fig 1C shows for each replicon the outline arrangement of elements characteristic of origin regions. In the main chromosome, c1, these features—DnaA boxes, short sequence repeats, IHF site, AT-rich block—are sufficiently dispersed to leave the precise position of the replication origin uncertain; the geography of this region is examined in further detail in the Supplementary Information (S1 Fig). The origin regions of the secondary chromosomes, c2 and c3, each include an ORF with strong similarity to *repA* genes that specify classic replication regulators of low copy-number plasmids, as well as typical clusters of 19–21 bp iterons to which these regulators bind, leaving no doubt that these replicons originated as plasmids (for detail, see S2 Fig and S1 Table).

Chemical assay of the amount of DNA in exponentially growing *Bcen* J2315showed it to be 1.6 genome equivalents per average cell (S2A Table). To verify the replicon copy numbers and to examine the basic replication parameters of the *Bcen* genome, we initially used quantitative Southern blot hybridization to measure the relative abundance of replication origins and termini. The data showed the relative concentrations of the chromosomal *ter* sequences to be close to unity (S4 Fig), which together with the DNA assay above confirmed that the basic copy number of each chromosome is one per cell; the higher value for the number of the plasmid *ter* sequences, 1.3–1.4, results from most plasmids completing replication during the first half of the cycle (see below).

**Fig 2:**
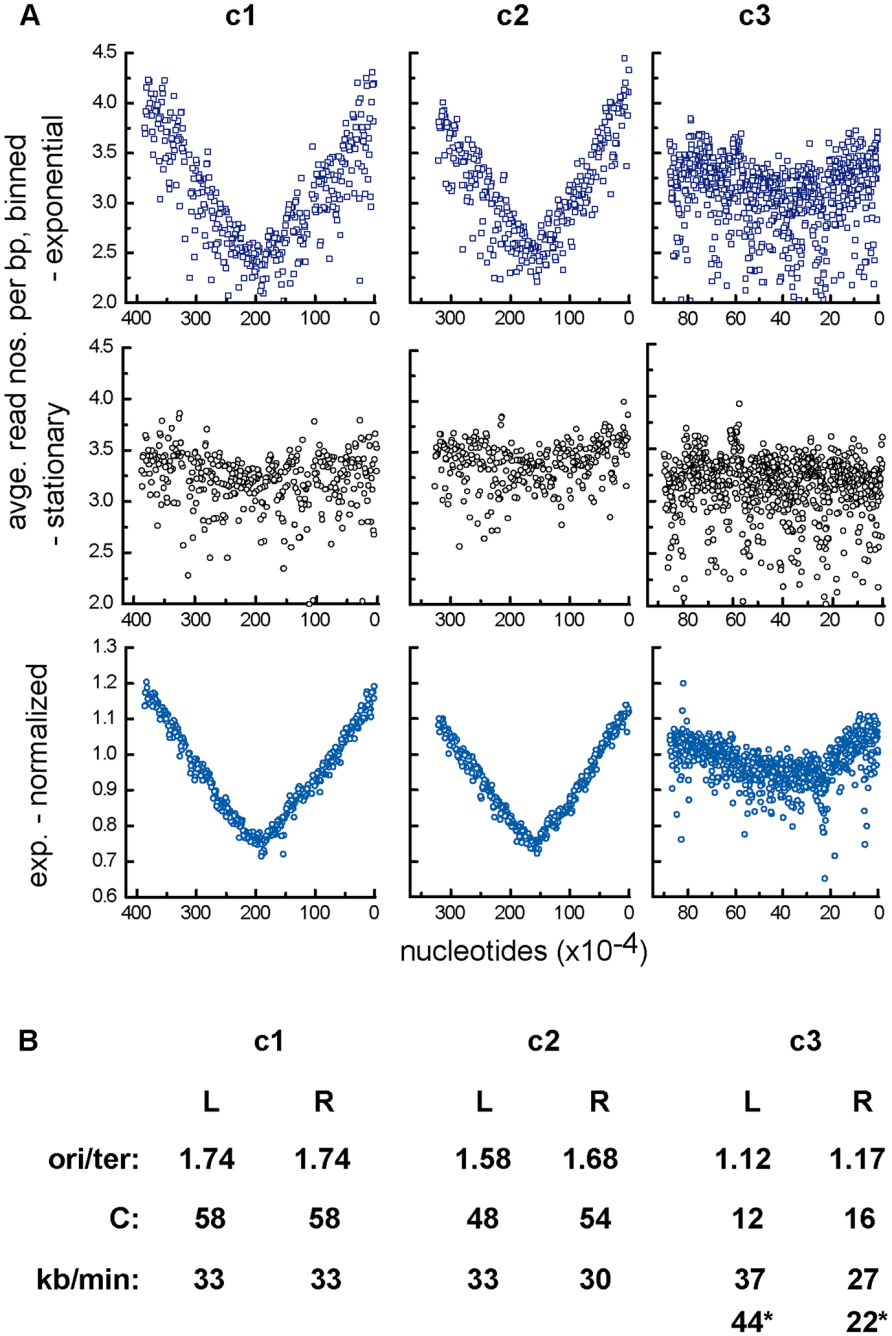
Replication characteristics determined by high-throughput sequencing. **A**. Base-pair frequency gradients. Data points are averages of binned read numbers representing successive blocks of 10kbp for c1 and c2 and 1kbp for c3. The top panels show the raw data for DNA from exponentially-growing cells, the third row shows the same data after division by the correspondingly binned data obtained from stationary-phase cells (second row). Nucleotide positions on the abscissa are reversed to conform to the intuitive sense of right and left chromosome arms. Because the data are plotted as raw read frequencies, relative copy numbers of the replicons can be read from the ordinates. **B**. Calculation of chromosome replication period, C, and speed. Origin/terminus ratios were used to calculate the time taken to replicate each chromosome arm from *ori*/*ter* = 2^C/τ^. In the case of c1 and c2, the concavity of the stationary-phase base-pair frequency curves would falsify calculation of origin/terminus ratios from normalized data, necessitating use of the raw data plot. For this, raw data *ori*- and *ter*-proximal points that corresponded to points intersected by the linear regression plot of the normalized data were connected by lines whose upper extremities and intersection were taken as the *ori* and *ter* values respectively. The c3 stationary-phase bp frequency curve is essentially flat, validating the normalized data. The * values for c3 replication speed are calculated on the basis of terminus displacement creating arms estimated to be 340 and 530 kb long.

The *ori*/*ter* ratios obtained from these data should allow us to confirm experimentally the strong indication from GC-disparity analyses (Fig 1; ref. 10) that replication of each chromosome is bi-directional, as well as to determine how long it takes (C period). However, anomalous hybridization behaviour of certain probes led us to use an alternative method, direct determination of base-pair frequency by sequencing. DNAs purified from Nel13 cells growing exponentially in LB (doubling time 72 mins), as well as from cells incubated in stationary phase for 8 hours to allow replication to terminate, were processed and subjected to deep sequencing (see Materials & Methods). Read-number data were binned and plotted as a function of chromosome position (Fig 2A). Scatter arising from variation in amplification and sequencing efficiency was minimized by normalizing the raw data (first row) with respect to the corresponding reads from stationary-phase cells (second row), yielding the relatively tidy curves shown in the third row.

The base-pair frequency gradients show replication of the main chromosome, c1, to proceed from the predicted origin region in both directions and to terminate diametrically opposite. Replication of c2 is essentially the same, although the fully symmetric pattern observed with c1 is not seen; origin-proximal sequences in the left arm are slightly less abundant than those on the right. Possible explanations for this asymmetry are occasional unidirectional, clockwise replication from the c2 origin or delayed initiation of the left arm. The replication mode of c3 is more complex. It appears to be predominantly bidirectional with a difference in frequency of the origin-flanking sequences implying sporadic unidirectional or delayed replication as suggested for c2. But in addition, the steepness and extent of the two gradients differ from each other. The base pair frequency minimum is displaced anticlockwise, such that the left and right replichores occupy, respectively, about 60% and 40% of the c3 replicon. This further asymmetry can be provisionally interpreted as resulting from relatively slow movement of the clockwise fork and from absence of a strong replication terminator opposite the origin, such that the anticlockwise fork terminates within the right chromosome half.

The bp frequency gradients were used to calculate the replication time, C, of each chromosome (strictly, each chromosome arm), from *ori / ter* = 2^C/τ^, (C = τ [log ^(ori^/_ter)_/ log2]; [27]) where τ = culture doubling time. Although the normalized frequency curves had enabled us to discern the overall replication pattern of c1 and c2, they proved unsuitable for quantitative purposes owing to the residual concavity in the corresponding stationary-phase curves used as references; this presumably reflects failure of some cells to terminate c1 and c2 replication even long after cessation of net growth. Accordingly, we estimated *ori* / *ter* ratios from the raw data plots (see legend), and the C values derived from them are shown in Fig 2B. For c1 and c2 these are 58 and 51 (average) minutes respectively, representing a replication speed of about 33 kb per minute, comparable to that of *E*.*coli* growing at the same rate (~36kb/min at τ = 72 min [28]).

In the case of c3 the essentially flat stationary-phase curve allowed normalization without distortion of *ori* / *ter* ratios. The data scatter and the shallow gradients render definition of the replication terminus approximate and prevent a similarly simple estimate of C. If the bp gradients indeed result from fast and slow forks meeting within the left half of c3, C would effectively be set by the slow fork, at about 16 minutes.

These times for the duration of c1 and c2 replication are readily accommodated within the ~75 minute cell cycle but cannot tell us when in the cycle replication is initiated. Direct observation of the positioning and separation of the replicated *ori* regions, although not a direct indicator of initiation, should enable us to address this question.

### Origin number and position during the cell cycle

To visualize the origin regions of the replicons in *Bcen* we used the binding of fluorescent derivatives of the native ParB proteins to their cognate *parS* clusters adjacent to each origin. We saw no indication that the ParB fusion proteins of c1 and c2 interfere with indigenous wt ParB function; indeed they showed partition activity equivalent to that of the native ParB proteins (S5A and S5B Fig), and none of the abnormalities stemming from deletion of *par* loci or provision of excess *parS* (see below). The fluorescent ParBc3 derivative was defective in partition function (S5C Fig) as well as aberrantly localized in the presence of wt ParBc3: to visualize the c3 origin region we used the phage P1 ParB-*parS* system of Li & Austin [29]

**Fig 3:**
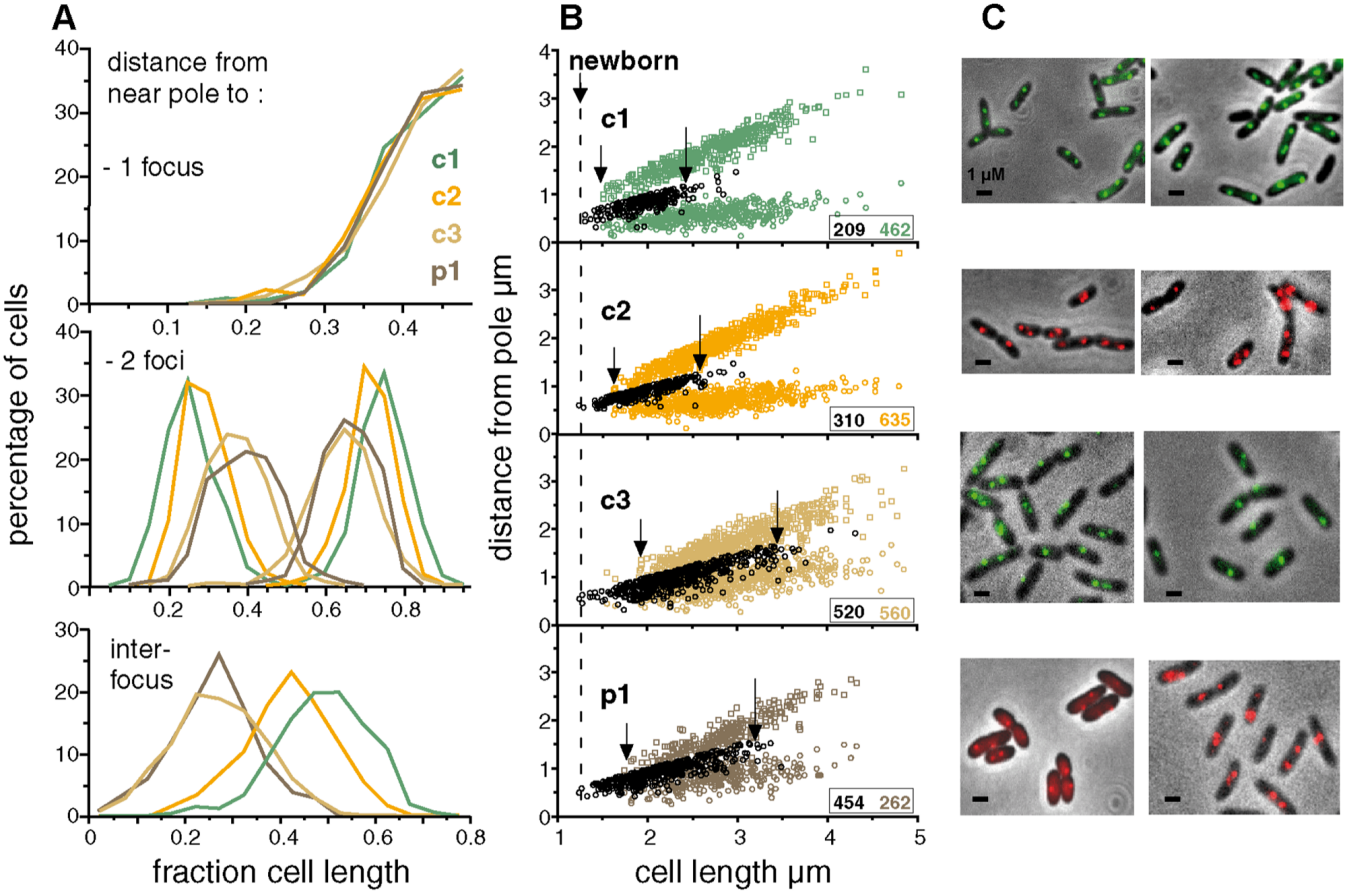
Position of origins relative to cell poles. **A**. Graphical summary of distances from the pole nearest a focus to the foci in one- and two-focus cells, and below, a plot of inter-focus distances. **B**. The positions of foci formed on *ori*-proximal sites by (**c1**) ParBc1::Gfp, (**c2**) ParBc2::Chfp, (**c3**) ParBP1::Gfp and (**p1**) ParB::Chfp were measured relative to the cell pole nearest a focus and plotted against cell length. Distances in cells with a single focus are shown as black-bordered circles in all cases; distances in cells with two foci are shown as coloured symbols. Arrows indicate the beginning of the two-focus clusters (left) and the end of the main one-focus clusters (right). Insets show numbers of cells scored, in corresponding colours. **C**. Examples of cells showing ParB::fp-marked origin regions. Scale bar is 1 μm.

Exponential-phase cultures of Nel13 derivatives, each carrying a pMLBAD plasmid from which one of the *parB*::*fp*s is expressed, were sampled for microscopic observation. In nearly all cells, whether grown in MGCC, LB or SOB medium, the three chromosomes were seen as a single centrally-located focus or as two foci positioned roughly symmetrically about the midpoint (Fig 3A and 3C), in agreement with the Southern hybridization results (S4 Fig). To estimate cell cycle parameters we examined the distribution of *ori*-proximal foci as a function of the length of cells growing at 30°C in MGCC with doubling times of ~110minutes (equivalent to ~76 mins at 37°C) (Fig 3B). The range of cell length over which replicated origins begin segregation is delimited, observationally, by the first appearance of two-focus cells and the end of the one-focus cell cluster, as arrowed. How these segregation events are placed within the cell cycle cannot be directly estimated from these data, but can be inferred on the assumption that the approximately two-fold range of newborn cell sizes reported for *E*. *coli* and *B*. *subtilis* [30–32] applies also to *Bcen* and read from the abscissa as 1.2–2.4 μm. The *ori* regions of c1 and c2 segregate within the length limits of 1.5- ~2.4 μm and 1.6- ~2.6 μm respectively. This behaviour indicates that segregation of c1 and c2 *ori*s begins early in the cell cycle and that it occurs within a range of cell lengths, ~1 μm, similar to the length range of newborn cells, implying close coupling with the cell cycle. It notable also that segregation of the plasmid-like origin of c2 is as tightly coupled as that of the c1 chromosomal origin.

Segregation of c3 origins is not seen until cells are 0.3–0.4 μm longer than the first to show c1 and c2 segregation, implying cell cycle phasing. However, its longer segregation range, 1.6 μm (1.9–3.5), suggests that any coupling of c3 segregation to the cycle is less strict than for the larger replicons. Plasmid p1segregation is first seen in cells ~0.2 μm shorter than the first to segregate c3; its length-at-segregation range, 1.5 μm (1.8–3.3), is similar to that of c3 and also indicates relatively loose coupling to the cycle.

These data are uninformative as to the time at which replication is initiated, but an indication of whether segregation follows initiation immediately or after a delay can be gleaned from DNA/cell data. Assuming that initiation at the c1 origin occurs at a fixed point in the cycle, as is the case for those bacterial chromosomes studied [14,33,34], and that initiation at the c2 origin is similarly phased with the cycle, as our data suggest (Fig 3, and see Discussion), we can calculate that replication of c1 and c2 is initiated shortly before or at the end of the preceding cycle, as derived in S2B Table, implying that a significant interval probably separates initiation and visible segregation of the c1 and c2 origins seen here.

Replotting of the 2-focus data as a fraction of cell length and as interfocal distances (Fig 3A) confirms two aspects of segregation behaviour: the widths of focus distribution are more restricted for c1 and c2 than for c3 and p1, implying more precise positioning of the former two, and the average distance moved towards the poles follows the order in which partition was initiated, indicating an order of segregation ages, c1 < c2 <p1≤ c3.

**Fig 4:**
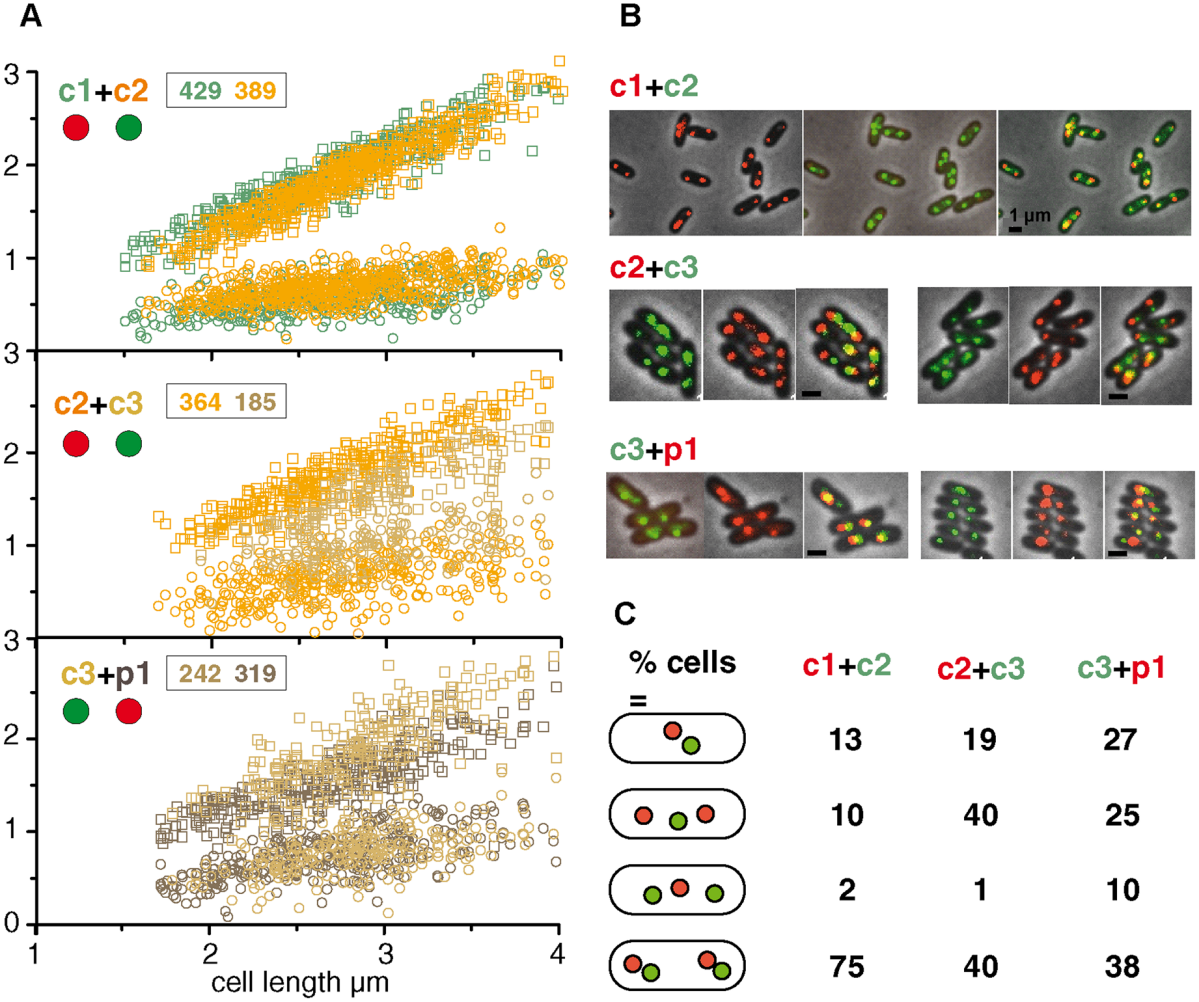
Positions of two origins visualized simultaneously. **A**. The origins of two replicons are plotted relative to the distance from the pole nearest any focus. Insets show numbers of cells with two foci of the replicon correspondingly coloured in the plot, comprising those with either one or two foci of the other replicon visualized; cells lacking foci of either replicon were excluded from the analysis. Red and green discs indicate respectively Chfp and Gfp fusions used to mark the origins shown. **B**. Examples of cells with origins of two replicons co-visualized. The separate components of the overlays are shown as examples of the images on which length measurements were made. Scale bar is 1 μm. **C**. Frequencies of focus combinations, shown as percentage of total cells scored.

The difference in distributions of c1 and c2 two-focus cells over the length range within which segregation occurs (1.5–2.6 μm, see above) was compatible with the order above but for c1 and c2 was at the limit of statistical significance (S6 Fig). Because it appeared possible that experimental variability arising from our use of independent cultures for visualizing each replicon could have reduced the reliability of comparative segregation times, we again measured focus positions, this time using comparison at the single cell level with pairs of replicons marked at their origins. The results shown in Fig 4A confirm that segregation of the c1 origin generally precedes that of c2, the c2 origin that of c3, and the p1 origin also, but more narrowly, that of c3; examples of the cells observed are shown in Fig 4B. These data also confirm the relative average destinations of segregated origins shown in Fig 3A. If the segregation order is correct it should be reflected in the relative frequency of focus combinations. The tabulation of cells in each focus category (Fig 4C) bears this out. Doubling of c1 foci generally precedes that of c2, c2 nearly always precedes c3, and on average p1 also precedes c3. These data can be used to estimate average cell age at segregation for each replicon (S2C Table). The order is not absolute, however. In particular, in a minority of the cells the c2 origins segregated before the c1 origins, behaviour which is concealed in the focus distributions. Possible explanations include a looser regulation of c2 initiation such that it occasionally precedes that of c1 and, more likely, an occasional prolonging of the c1 initiation-segregation interval.

Having defined in outline the main features of the *Bcen* cell cycle, and knowing that the ParABS systems of each replicon can act independently and specifically to partition plasmids in *E*.*coli* [10,21], we next asked whether the Par systems also behave this way in their natural host, where a possible involvement in other processes might influence the coordination of segregation.

### Impact of inactivating ParABS systems

Mutation of chromosomal ParABS systems do not only impair segregation but also affect replication, DNA compaction, cell division and viability [19,35–39] through direct, functional interaction of Par proteins with the regulators of these processes, e.g. the initiator, DnaA [11,13], the condensin, SMC [40,41], the division inhibitor, MipZ [42]. We explored the range of roles that the *Bcen* Par systems play by observing the effects of nullifying each system on growth, morphology, replication and partition. ParAB function was disrupted in two ways: by deletion of *parA* or *parAB* from each replicon, and by introduction of excess *parS* sites to deplete ParB available to the chromosomal *parS*s (see Materials & Methods). The deletion in the *ΔA*c1mutant is polar on *parB*, reducing the ParB protein level to <5%that of wild type (S7 Fig) and rendering this strain phenotypically ParAB-minus. Excess *parS* sites were introduced either singly or as the natural cluster (for c2, c3 and p1) on the vector pMMBΔ (10–15 copies per cell). To obtain reproducible growth of and focus formation by mutant cells it was necessary to use growth media other than the MGCC used so far, as noted in the figure legends.

#### *oriC* positioning

**Fig 5:**
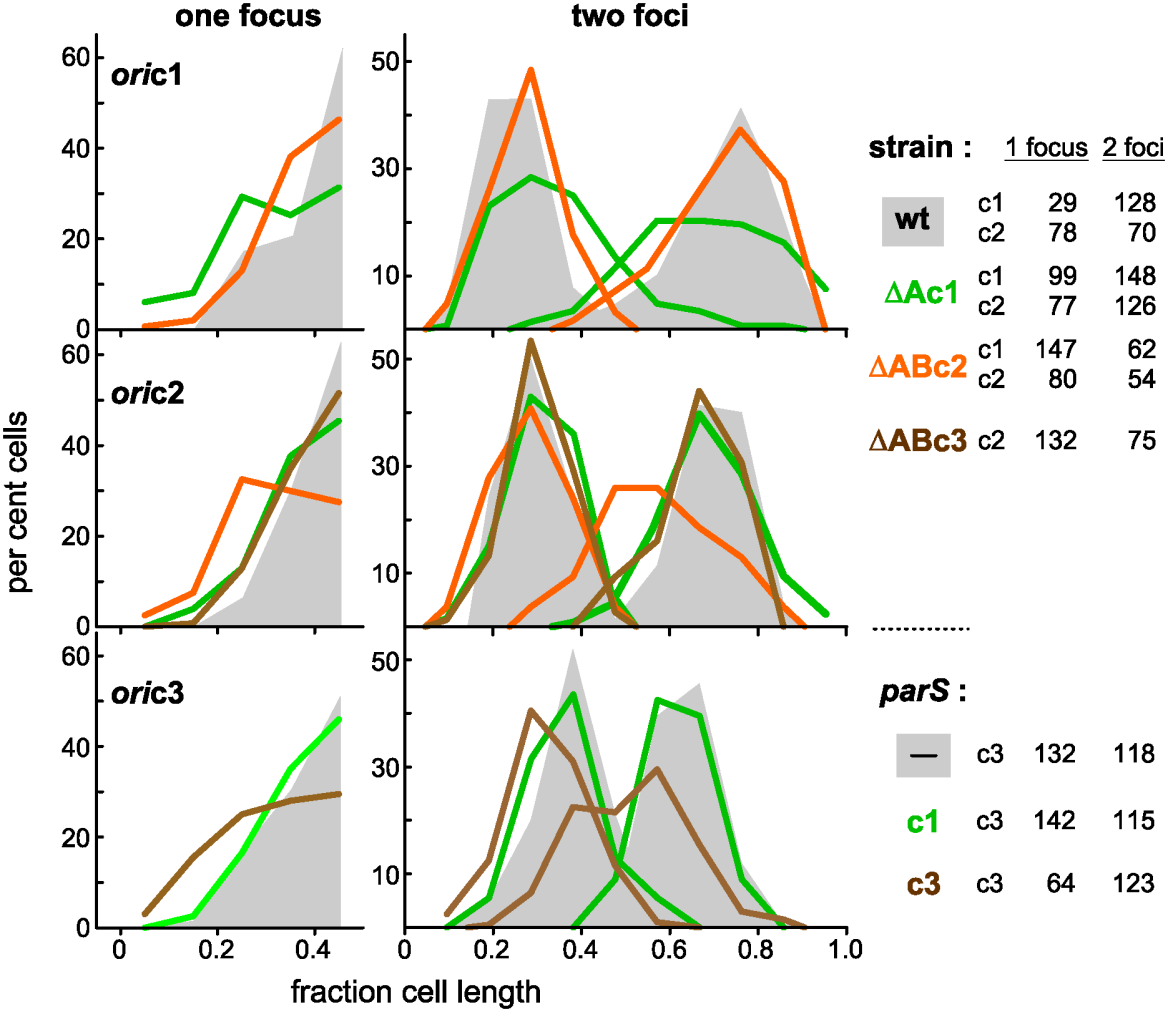
Effect of ParABS disruption on distribution of replication origins. The chromosome origins of Δ*par* and corresponding wild type strains were marked with ParB::Fp proteins, and the distances of fluorescent foci from the most focus-proximal pole were measured and binned in intervals of one-tenth cell length. The ParABS disrupting factors are shown at the right: deletion mutations in *ori*c1- and *ori*c2-marked cells (grown in MglyC), plasmid-borne *parS* sites in *ori*c3-marked cells (grown in SOB). Origin distributions in cells with undisturbed Par function are shown as shaded areas. Numbers of one- and two-focus cells analyzed are shown at the right.

The effect of Par disruption on the positioning of the three chromosomal origins is shown in Fig 5. Inability to introduce *parS*P1 into the Δ*parAB*c3 mutant for marking *ori*c3 obliged us to use excess *parS*c3 instead. For each chromosome, the distribution of the origin cognate to the disrupted Par system was broader and, in two-focus cells, the separation of origin copies reduced. Minor deviations in positioning of non-cognate chromosomes from the wild type pattern, e.g. *ori*c1 in Δ*AB*c2 cells (top panel) and *ori*c2 in Δ*Ac1* cells (second panel), could result, at least partly, from general adjustments of nucleoid disposition to altered behaviour of the cognate chromosome. We can conclude that the three chromosomal Par systems are indeed necessary for effective segregation of their respective chromosome origins. It is also clear that none of them has a major influence on segregation of the others, at least of their origin domains, meaning that partition of each of the *Bcen* chromosomes is operationally independent.

#### Replication control

**Fig 6:**
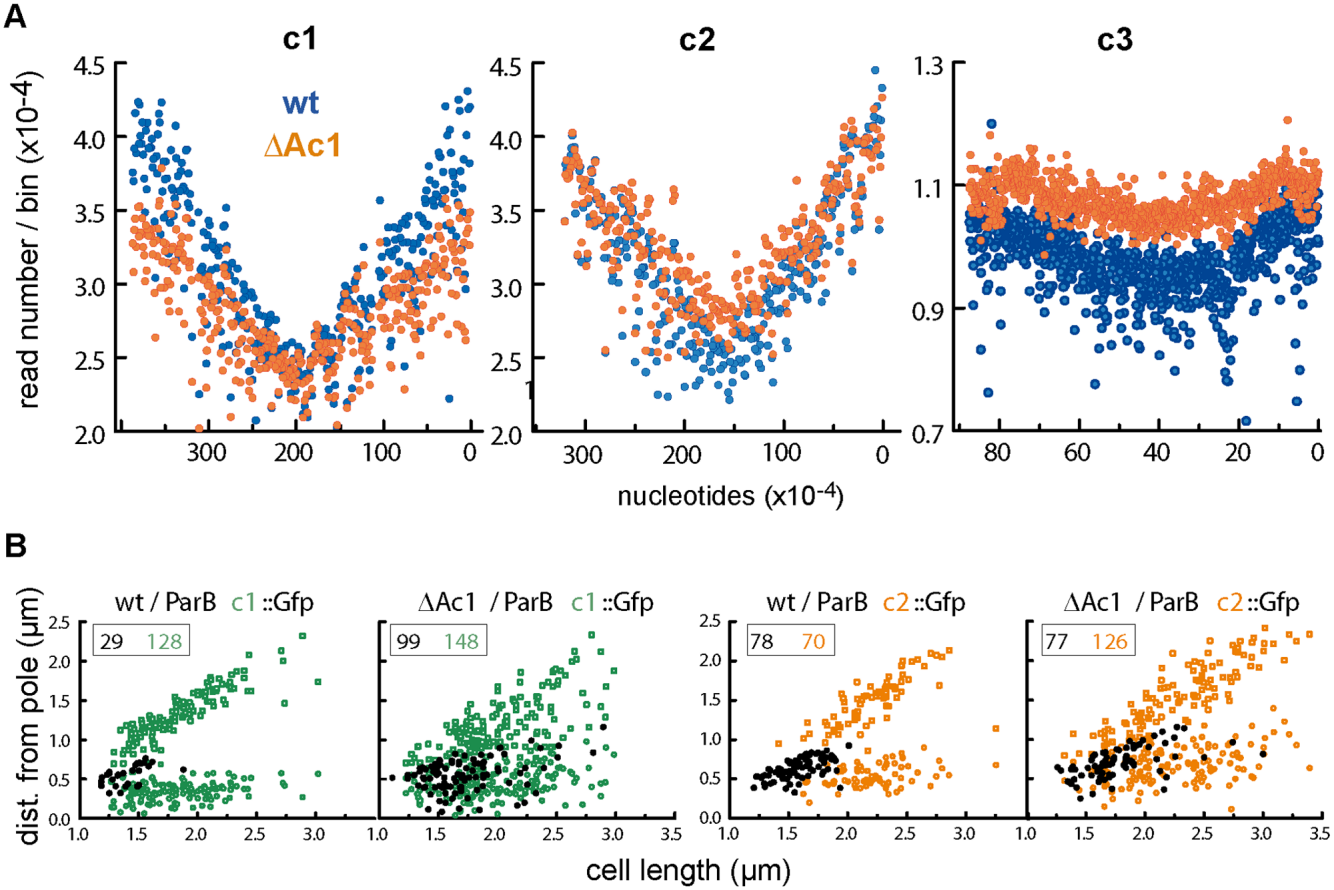
Replication characteristics of chromosomes in Δ*parA*c1 mutant cells. **A**. DNA purified from exponentially-growing FBP47 (Nel13 Δ*parA*c1) cells was purified, processed and analyzed in parallel to wild type (Nel13) DNA, as outlined in the Fig 2 legend. The FBP47 data are shown in orange superimposed on the Nel13 data in blue (transposed from Fig 2). Raw read numbers are shown for c1 and c2: these reflect relative quantities of loci within a given strain but not of a given replicon between wt and mutant strains. **B**. Positions of c1 and c2 origins in wild type and Δ*Ac1* mutant cells growing in MglyC medium at 30°C. Pole-to-focus distances are shown black for one-focus cells and coloured for two-focus cells, as in Fig 3. In these conditions, cells grow more slowly (τ ~140 mins) than in the previous analyses of focus position shown in Figs 3 and 4 (τ ~110 mins), and the cells are correspondingly smaller.

To test whether the ParABS system of c1 contributes to regulating initiation of replication, as do its orthologues in *B*. *subtilis* and *V*. *cholerae* [11–13], we determined the base-pair gradients of chromosomal DNA extracted from exponentially-growing Δ*parA*c1 cells, using deep sequencing as for the wild type, above. Fig 6A shows bp frequencies (raw for c1 and c2, normalized for c3, as in Fig 2) arrayed along the length of the three chromosomes. The mutation affects the profiles of all the chromosomes, chiefly by reducing the bp gradients. The *ori/ter* ratio of c1 falls from 1.74 to 1.46 and of c2 from 1.68 to 1.46 on the right arm and 1.58 to 1.38 on the left. The C periods derived from these *ori/ter* ratios and the longer mutant generation time are higher than for wt—in the case of c1 C rises from 58 mins to 74 mins, and of c2 from 54 mins to 74mins (right), 63 mins (left)—but constitute a smaller fraction of the cell cycle (for c1, 0.81 of 72 mins vs 0.54 of 136 mins), suggesting a higher replication speed relative to growth rate in the mutant. The c3 ratios are also reduced, although the data scatter makes quantitative comparison difficult, as do the disappearance of the asymmetry seen in the wt profile and the appearance of twin peaks in the bp frequency profile ~120 kb either side of the c3 origin region, rather than a single peak coinciding with it. Indeed this latter feature suggests that c1 ParA is a determinant of the mode of initiation, in addition to affecting its timing.

The significantly reduced *ori*/*ter* ratios are consistent with the mutant initiating replication of the three chromosomes later in the cell cycle than wt, and partly compensating with a higher rate of fork movement (relative to growth rate); compensating elongation rates have been reported to occur in *E*. *coli* mutants with altered initiation timing [43,44]. However, while the lack of *parA*(*B*) c1 appears to subject the three chromosomes to roughly equal reductions in replication speed, the drop in read frequencies of c1 sequences is much more marked than that of c2 and c3, indicating a reduction in the fraction of total DNA represented by the main chromosome. This implies that c1 replication is delayed as a result of the mutation. The persistence of single-focus Δ*Ac1* cells fluorescently-marked at *ori*c1 and *ori*c2 (Fig 6B) is consistent with frequent delay of initiation, although retarded segregation presumably contributes also. Some measure of this is provided by comparison of two-focus cells: in the case of *ori*c1 not only is segregation delayed but separation of origin replicas is inefficient, resulting in a scattered focus pattern rather than the two discrete groups seen in wt cells, but while segregation of *ori*c2 is also delayed, the presence of the functional c2 Par system results in the separation of *ori*c2 replicas being almost as efficient as in *parA*c1^+^ cells.

These results, together with the known functional interaction of ParA family proteins with DnaA [11,13] suggest that the c1 ParABS system plays an important role in regulating initiation of its own chromosome.

#### Growth, fitness and morphology

**Fig 7:**
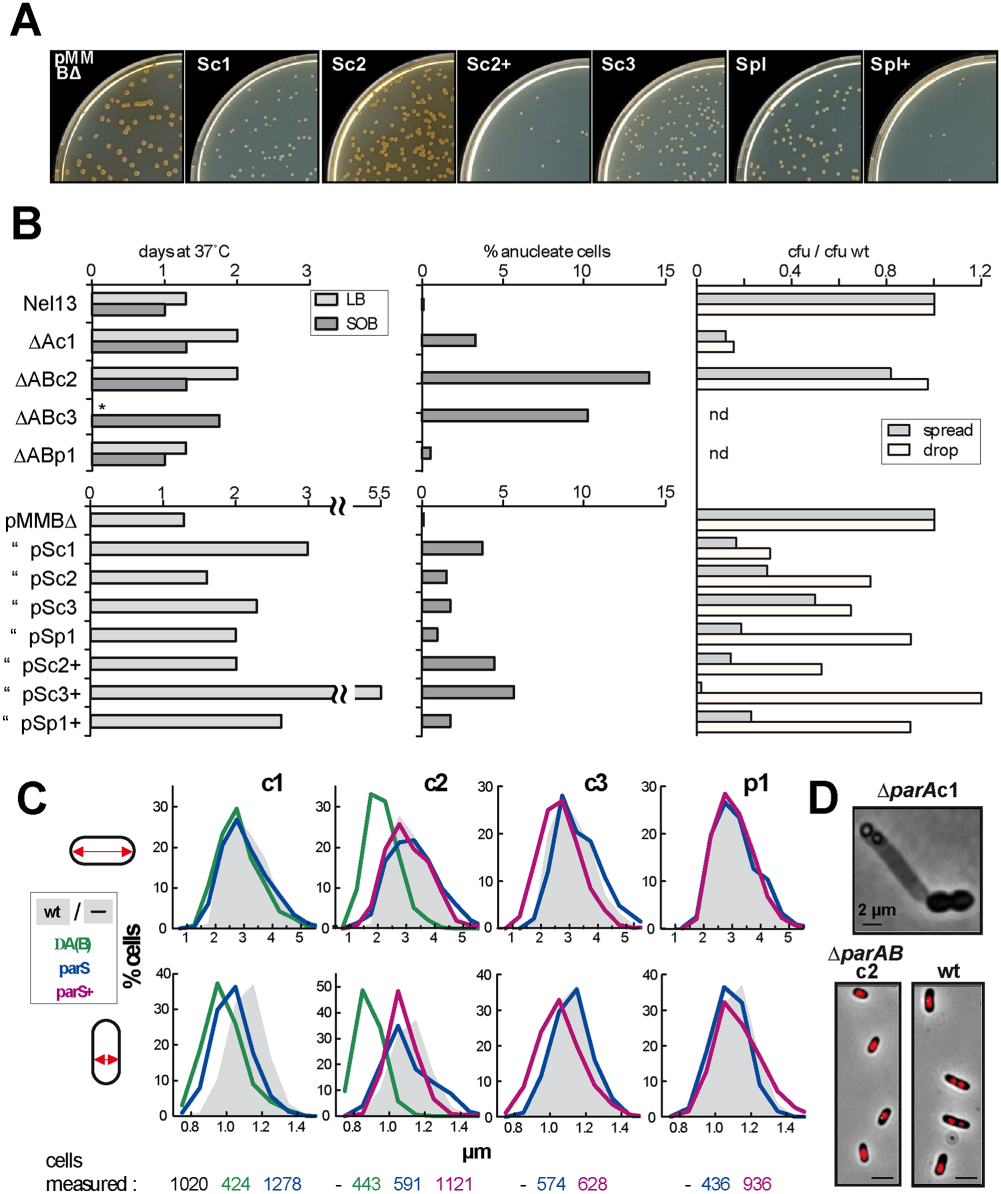
Role of Par systems in cell growth and morphology. **A**. Size of colonies formed at 3 days from cells transformed by plasmids carrying the *parS* sites indicated, either as single sites (e.g. Sc2) or as the natural clusters (e.g. Sc2+). *Bcen* makes pigment as cells enter stationary phase: faster-growing colonies are thus brown, slower-growing colonies still white. **B: left panels** Time of appearance of colonies after spreading on the agar media shown. Colonies were scored every 8 hours until the count reached its maximum; **middle panels** Cells grown exponentially in SOB were fixed, stained with DAPI and scored by fluorescence microscopy; **right panels** Viability on solid media. Cells grown in SOB to OD_600_ ~1.0 were diluted and plated on LB agar either as a 0.1ml sample spread with glass beads or as a 10μl drop. The numbers of colonies are expressed as the ratio to the wild type colony count corrected for differences in OD. **C**. Cell dimensions. Cells growing exponentially in SOB at 37°C were fixed for viewing by phase contrast microscopy and their length (top panels) and width (bottom panels) were measured. The reference strains for the *ΔA(B)* mutants and *parS*-transformants were Nel13 without and with pMMBΔ respectively. **D**. Illustrative examples of abnormal cell phenotypes characteristic of mutants Δ*parA*c1 (top, and see S8 Fig) and Δ*parAB*c2 (bottom). The nucleoids of the Δ*parAB*c2 cells are revealed by DAPI staining. Scale bars are 2 μm.

All disruptions of c1, c2 and c3 partition decreased growth rate, from 70–80 minutes in LB at 37°C to 100–140 minutes. However, doubling times in liquid medium were not reproducible, presumably owing to randomly-arising mutations that suppress Δ*par*-induced growth defects, and subsequent overgrowth by the mutants. For comparative purposes we adopted a more reliable parameter, used previously [21] for estimating *parS*-induced growth inhibition—delay in colony appearance on solid medium. Fig 7A illustrates the reduced size of colonies formed from cells transformed with plasmids carrying *parS* sites, and Fig 7B (left panels) shows the longer delay in appearance of colonies formed from mutant and *parS*-transformed cells. The *parAB* deletions in c1, c2 and c3 all retard growth, that in c3 most severely; indeed the deletion in c3 prevented growth on LB medium. *ParS*-mediated interference showed corresponding behaviour, that of c3 again being the strongest and causing a delay in colony appearance of four days relative to wild type. The case of the p1 plasmid is less clear-cut: whereas deletion of its *parAB* does not affect growth, introduction of supernumerary *parS* sites causes a lengthy delay in colony appearance (Fig 7B, pSp1^+^). Introduction of chromosomal *parS* sites also causes growth delays more pronounced than those of the corresponding *par* mutants, consistent with the notion that these recently-transformed cells do not carry suppressor mutations that might alleviate the mutants' growth defects.

All *parAB* deletions generate anucleate cells (Fig 7B, upper middle). Interference by *parS*, including that of p1, also resulted in anucleate cell accumulation, more strongly with the full *parS* complements of c2, c3 and p1 (lower middle). Loss of a specific chromosome is to be expected upon disruption of its Par system. Loss of the entire genome suggests that absence of any one chromosome provokes either radical mis-segregation of the others, particularly following changes in nucleoid bulk upon loss of c1 or c2 or, in the cases of the smaller replicons c3 and p1, generalized DNA degradation induced by a toxin such as CcdB [45].

Cell viability was measured as colony formation by cells applied to the agar medium surface either by spreading with glass beads or as a drop (Fig 7B, right-hand panels); the former is detrimental to fragile cells, the latter protective. Only 10–20% of Δ*Ac1* or *parS*c1-transformant cells formed colonies when spread, a fraction little increased by drop application, implying that most anucleate cells originating in these strains are very fragile and lyse quickly in liquid, leaving relatively few to be scored as DAPI-negative upon viewing under the microscope (Fig 7B, central panels); given its evident fragility the particular triplet cell morphology (Fig 7D and S8 Fig) might arise more frequently than the ~10% of cells observed to exhibit it. In contrast, most Δ*AB2*cells form colonies when spread. Their relative robustness could account for the persistence of the anucleate cell fraction. Cells transformed with the c2, c3 and p1 *parS* sites also appear less viable when spread, but drop sample counts indicate a level of viability > 50%, implying that unlike abrogation of *parA*c1 these Par system disturbances weaken the cells but inflict only limited mortality.

A further effect of Par disruption was reduction in cell size (Fig 7C and 7D). Δ*ABc2* cells showed the greatest reduction, with an average length 0.75x and width 0.8x wild type. Δ*Ac1* and *parS*c3-transformed cells were also smaller, though to a lesser degree. Reduced cell-size is a normal consequence of lower growth rate, but the greater reduction of Δ*ABc2* cells appears to be a phenotype particular to this Par system.

## Discussion

### Segregation pattern and the cell cycle

**Fig 8:**
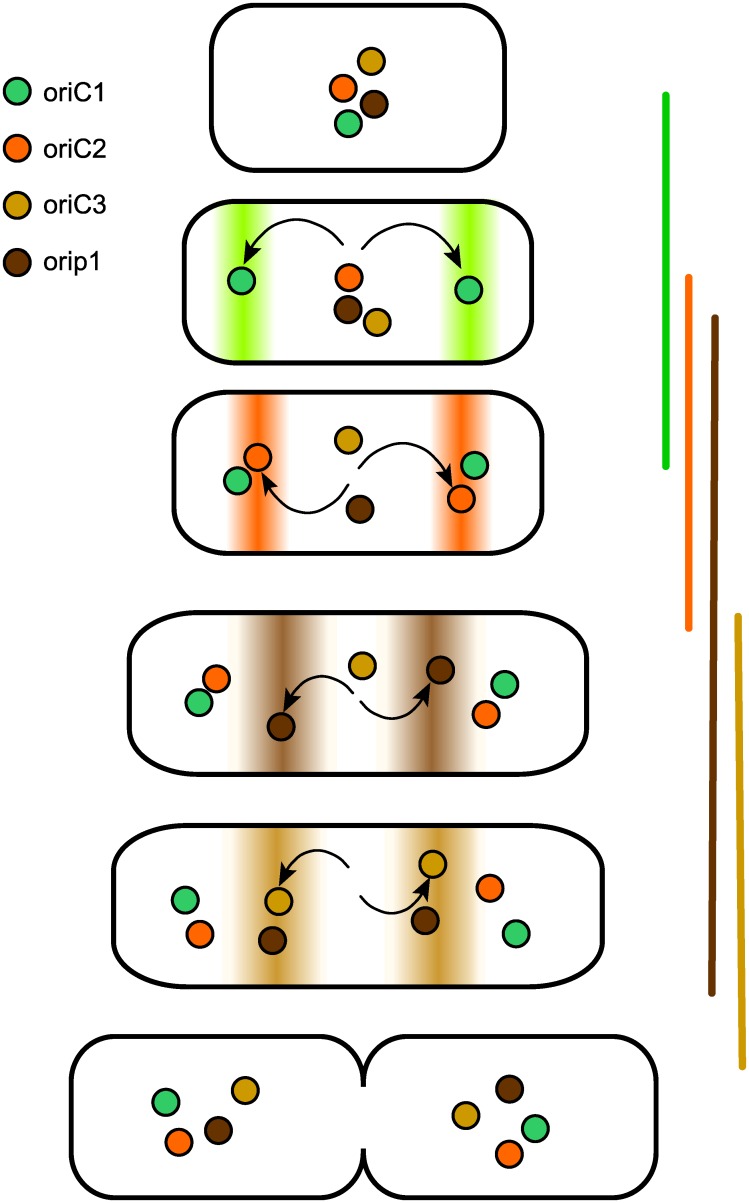
Segregation and positioning of replication origin regions during the *Bcen* cell cycle. Sequential partition in the order c1 –c2 –c3 –p1 is depicted. All origins are shown as gravitating to the centre of cell halves before division, as deduced from their central position in single-focus cells.

Our aim in undertaking this study has been to understand how bacteria with split genomes organize the maintenance of their multiple replicons within the cell cycle—how they time replication and segregation of plasmid-like chromosomes to avoid division delays and how they programme partition to avoid entanglement. For *B*. *cenocepacia* J2315, the results obtained point to a basic strategy—successive activation of the replication and segregation of each origin from a single locale, the cell midpoint (Fig 8). This maintenance mode resembles that of the only other multipartite genome for which these issues have been studied, that of *V*. *cholerae*, insofar as segregation of replicon copies is staggered through the cycle, but differs from it in that the resting origins of *V*. *cholerae* Chr1 and Chr2 are physically distant from each other, at the cell pole and midpoint respectively. The *Bcen* succession proceeds from segregation of the primary chromosome early in the cycle to that of secondary chromosomec2 shortly afterwards and then of c3 and the plasmid p1 at later, less well-defined cell ages (Figs 3 and 4). Notably, the immediate destinations of the replicated origins follow this order of segregation (Fig 3A), and only at around the time of cell division do all origins assume the midcell location seen in single-focus cells. Time-lapse monitoring of *ori* and *ter* movement will be needed to define this repositioning in more detail.

Although we have presented the temporal and spatial aspects of multi-replicon maintenance as separate, for *Bcen* they may be intimately related. In a newborn daughter cell where all four *ori-par* regions are close together at midcell, attempts at simultaneous segregation could be self-defeating. The ParA proteins of all four systems are of the Walker-box ATPase type, like those of the F and P1 plasmids and of bacterial chromosomes which appear to use the nucleoid surface to modulate transitions in ParA conformation essential to the partition mechanism [46–49]. It is unclear whether two partition processes of this type can simultaneously use overlapping nucleoid patches and move their origins over them. Staggering partition of the three large replicons should help avoid such scenarios of physical interference, thus improving partition efficiency. Participation of the c1 Par system in this temporal separation is suggested by the apparent delay in c1initiation timing and altered c3 bp frequency gradients in the Δ*parA*c1strain (Fig 6). The precedents for functional interaction of ParA proteins with DnaA [11,13] and the presence of DnaA-box clusters in the *Bcen* chromosomal origins lends credence to this proposal.

A subsidiary aim of this study was to verify that the specificity of action which each Par system had manifested previously in *E*. *coli* and *in vitro* [10,21] applied also in the systems' native cells. The importance of this verification was underlined by our discovery of overlapping specificities in other Burkholderia species [21]. The defects in origin positioning that appeared only in cells carrying the cognate *parA(B)* deletion (Fig 5) confirmed that this was so. It is unlikely that the *par* systems alone are primarily responsible for timing the segregation of replicated origins. Rather, it is through their regulatory role in initiation, indicated by changes of bp frequency gradients in the Δ*Ac1*mutant (Fig 6) that they could contribute to replication timing. The distinction between global effects on initiation and a specific role in partition is mirrored at the temporal level—our preliminary estimate of initiation age suggests an interval amounting to ~15% of the cycle between initiation and origin segregation (S2B Table). Even if future work proves this accurate, it applies only to c1 and c2, the replicons that contribute significantly to the genome mass on which the calculation is based. We have no results that bear on whether newly replicated c3 and p1 origins remain colocalized or cohered for a period before segregation. We do not know, for example, whether clustering of p1 siblings, a phenomenon held responsible for sub-copy number focus numbers of several *E*.*coli* plasmids [50], artificially prolongs the apparent age at segregation of p1 seen in Fig 3.

### Status of secondary chromosomes

It is reasonable to question the term "chromosome" as a title for large secondary replicons. In the case of *Bcen* c2 and c3 the issue is not settled. First, their complement of essential genes is very limited [20], suggesting that acquiring a few of them ensured that the replicon became indispensable and removed any selective pressure to accumulate more. Moreover, none of the acquired core genes is a constant feature of secondary chromosomes, as would be expected if the replicons were chromosomal in the eukaryotic sense. Second, and more importantly in the present context, the replication control systems resemble those of plasmids with a specific initiation regulator and iteron-like binding sites rather than that of primary chromosomes, for which the near-universal DnaA acts as the main regulator. Likewise, the *parABS* partition systems are variable and specific rather than based on the "universal centromere" [51] of the main chromosome. To reflect these characteristics, as well as necessity for cell viability and a GC content close to that of the chromosome, the term "chromid" has been proposed as a replacement for the often ambiguous labels—secondary chromosome, megaplasmid, etc—in use till now [52]. This sensible proposal appears at first sight applicable to c2 and c3. Nevertheless, certain criteria were not taken into account in the definition of chromids. The asymmetry of KOPS distribution centred on a *dif* site (Fig 1) is characteristic of chromosomes. Likewise, linkage to the cell cycle can reasonably be considered a chromosomal attribute, and has been in the case of *V*. *cholerae* Chr2 [14]. As pointed out above, a replicon size comparable to that of the main chromosome obliges cycle-phased replication, and in this sense is a chromosomal characteristic. Our data (Figs 3 and 4) indicate that in general, segregation, and presumably the prior replication, of c2 are as well phased with the cell cycle as they are for c1. On this basis we propose that the c2 replicon of *Bcen* J2315 qualifies as a chromosome. The c3 replicon, on the other hand, does not, since its segregation is only loosely timed with respect to the cycle. Moreover, its essentiality is unclear. Agnoli et al [53,54] obtained from many isolates, representing16 species of the 17 in the *B*. *cepacia* complex, derivatives cured of c3 whose growth properties were essentially unchanged, demonstrating that Burkholderia c3 replicons are in general neither chromosomes nor chromids but simply plasmids. However, *B*.*cenocepacia* J2315 was not among these species. The status of its c3 replicon has still to be determined.

Whether or not synchronization of replication with the cell cycle justifies elevation of c2 to chromosome status, the question of how such coupling came about is important. The simplest answer would be that an inherently synchronized plasmid was the progenitor of the present-day c2; the view that plasmids replicate at random through the cell cycle is based on experiments performed on only a few *E*. *coli* plasmids [6–9], which might not be representative of the plasmid universe. Alternatively, a synchronizing host-plasmid interaction might have been selected once a c2 ancestor, already essential, had expanded to a size problematic for the cell cycle [55]. A further possibility is linkage to replication of the c1 chromosome via a common regulator. Inspection of the c1 and c2 *ori* regions (S1 and S2 Figs) provides no obvious evidence for shared or overlapping regulatory processes (apart from the purely speculative roles of the clustered 7mers). A synchrony element of this type has recently been discovered in the *V*.*cholerae* genome [56]. A 70bp sequence situated 0.8kb from the origin on one 1.5kb arm of the primary chromosome (Chr1) was found to modify the replication regulator protein RctB of Chr2 in such a way as to stimulate Chr2 replication. Doubling of the 70bp element by replication was proposed to trigger Chr2 initiation, thus bringing Chr2 replication timing under the ultimate control of DnaA and coordinating it with the cycle. The possibility that an analogous mechanism links c1 and c2 replication in *Bcen* is worth exploring, although the observation that c2 foci occasionally double before c1 (Fig 4) suggests that such a mechanism is not an absolute requirement.

Although the c2 and c3 replicons appear to have evolved beyond the simple plasmid state, some of our data betray persistence of typical plasmid-like behaviour. Most striking is the asymmetry seen in the left and right arm bp frequency profiles (Fig 2). We favour the idea that this asymmetry reflects frequent failure of bidirectionality, such that c2 and c3 occasionally replicate by reverting to the unidirectional replication mode that presumably characterized their plasmid ancestors [57]. This observation has an important corollary—that a crucial component of the transition of a replicon from low-copy number plasmid to chromosome lifestyle is acquisition of the ability to replicate bidirectionally, thus halving the replication time and allowing the progressively expanding replicon to be replicated within the cell cycle. The alternative of rephasing initiation to allow it on not-yet terminated replicons, as seen in rapidly-growing *E*. *coli*, might not be compatible with an essentially iteron-based replication control system.

### Wider role of *parABS*

Apart from the demonstrated necessity of each replicon's ParABS system for its own partition (Fig 5), the appearance of several phenotypes specific to one or other of the disturbed Par systems suggests wider involvement in cell processes. Perhaps the clearest evidence for this is the altered replication of c1 and c3 in the mutant lacking the c1 ParA and ParB proteins (Fig 6). It suggests that in *Bcen*, as in *B*.*subtilis* and *V*.*cholerae*, the main chromosomal Par system helps regulate initiation. A further abnormality hinting at an expanded role for this system is displayed by cultures of cells in which c1 ParB is depleted by deletion of ParA or by *parS*c1 sequestration. About 5% of the cells form a triplet, one of whose terminal cells decondenses its nucleoid, elongates and eventually bursts, while the nucleoids of the two normal-sized partners are mis-positioned and show some lesser degree of compaction anomaly (S8 Fig). The mis-segregation and decompaction are reminiscent of the failure to load the condensin SMC at the *B*. *subtilis* and *S*.*pneumoniae*r eplication origins upon depletion of their ParB proteins [40,41,58], and suggests that the c1 Par system functions likewise in *Bcen*, a Gram-negative species.

Deletion of the c2 *parAB* operon also resulted in a specific phenotype, the reduction in average length and width of cells to about 70% of normal dimensions, and thus to an average cytoplasmic volume about one-third that of wild-type. Such contraction of the space available to the nucleoid might increase segregation deficiency beyond that specifically due to failure of c2 partition and contribute to the high level of anucleate cells generated in Δ*parAB*c2 cultures (Fig 7B). Participation of *parABS*c2 in regulating c2 initiation, analogous to that reported for the Chr2 chromosome of *V*.*cholerae* [39,59], might also contribute. *ParS*c2 interference does not produce the phenotype, implying that ParAc2 influences the mechanisms governing cell size or division.

Disruption of all chromosomal Par systems retarded growth, but loss of c3 Par function was particularly severe. The *parAB* deletion abolished growth on LB medium and imposed the longest colony-appearance delay on SOB medium, while the full *parS*c3 locus provoked a delay twice as long as the next most severe (*parS*c1; Fig 7B) as well as high cell fragility. These observations suggest a specific effect of *parABS*c3 on cell physiology. However, our data do not allow us at present to distinguish clearly between direct implication of *parABS* in host processes and inhibition of these processes by toxin-antitoxin system activation following c3 mis-segregation. All *Bcen* replicons carry toxin-antidote modules [54], and several of those in c3 appear important for the stability of their own replicons [53]. How these might account for the growth deficiencies seen here remains to be analyzed. Such TA mechanisms could underlie the dramatic loss of cell integrity that follows failure of Chr2 segregation in *V*.*cholerae* [59].

Defining the roles of the c2 and c3 Par systems in cell growth and morphology, as well as exploring their involvement in the cell cycle, is one of two major tracks towards elucidation of genome management in *Bcen* indicated by this study. The other is investigation of the mechanisms that enable the c1 Par system to act specifically in partition of its own chromosome and generally in regulating initiation of replication of all three chromosomes. Probing these aspects should throw light on the reciprocal adaptations that enabled ancestral cells and progenitor plasmids to evolve towards the multipartite genome states we now observe.

## Materials and Methods

### Strains

*E*.*coli* strain DH10B [60] was used as the primary transformation recipient for plasmid construction, and the *dam dcm* strain SCS110 (Stratagene) for propagating plasmids destined for *Bcn*. The basic Burkholderia isolate is *B*.*cenocepacia* J2315, genomovar III of the ET12 lineage, used as the UK cystic fibrosis reference strain [20]. The antibiotic-sensitive derivative, Nel13,was used for most experiments; instances of J2315 use are noted. Nel13 was obtained by deletion from J2315 c1 of a *mexAB-oprM* locus (*mex1*) that encodes an RND efflux pump (see ref. 61, where the strain is called ΔMex1). Deletion of the *par* loci was carried out by allele replacement: cells transformed with suicide vectors carrying the desired deletion were screened for loss of the integrated-then-excised vector by antibiotic sensitivity and of *parA(B)* by PCR (see S3 Table for details). The same approach was used to insert the *parS* site of phage P1near the c3 origin, as described [61], yielding strain Nel35.

### Plasmids

Plasmids used to produce fluorescent fusion derivatives of ParB proteins were constructed by first inserting the GFP and mCherry coding sequences (*gfp* and *chfp*respectively), tailed at their 5' ends by an *Nde*I site, into the *Sma*I site downstream of p*araBAD* in pMLBAD [62]. The *parB* genes of the four *Bcen* replicons were then amplified using primers with *Eco*RI and *Nde*I ends, enabling in-frame fusion to *gfp* and *chfp* in the pMLBAD vectors; the *parB*p1 gene had been mutated to remove the internal *parS*p1 site. For marking the *parS*P1 site in Nel35, the *gfp*::*parB*P1fragment cut from pALA2705 with *Bsr*BI and *Hin*dIII was inserted between the *Sma*I and *Hin*dIII sites of pMLBAD, to make pDAG825. Plasmids expressing tandem *parB*::*fp* genes were made by inserting *Nhe*I-*Hin*dIII fragments carrying one between the *Xba*I and *Hin*dIII sites in plasmids carrying the other, giving pDAG845 (*parB*c1::*chfp*-*parB*c2::*gfp*), pDAG846 (*gfp*::*parB*P1-*parB*c2::*chfp*), and pDAG847 (*gfp*::*parB*P1-*parB*p1::*chfp*).

Plasmids for providing excess *parS* sites were made as described [21], by replacing the *Eco*RI-*Mlu*I and *Apa*I-*Hpa*I fragments of *lacI* in pMMB206 [63] with fragments carrying single *parS* sites and *parS* clusters respectively. The control plasmid, pMMBΔ, is deleted of the *Eco*RI-*Mlu*I *lac*I fragment, which inhibits *Bcen* growth.

### Media and growth conditions

Media used were MGCC, composed of M9 salts (0.42M Na_2_HPO_4_, 0.22M KH_2_PO_4_, 0.009M NaCl, 0.018M NH_4_Cl, 1mM MgSO_4_, 0.1mM CaCl_2_), 3.4mM Na_3_citrate, 0.1% glucose, 0.2% Casamino acids, 0.04% tryptophan; MglyC, being MGCC with glycerol substituted for glucose; Luria-Bertani (LB) medium (1% NaCl version); and SOB. As an anti-contamination measure, media were routinely supplemented with gentamicin at10μg/ml, a concentration which does not affect *Bcen* growth. Antibiotics for selecting entry of plasmids into Nel13 and J2315 were used at, respectively, (μg/ml) chloramphenicol 40, 80; trimethoprim 200, 600; tetracycline 200, 400. Cultures were grown with aeration at 37°C or, for fluorescence microscopy, at 30°C.

### Growth rate and viability measurement

Growth rate in liquid medium was determined by periodic measurement of the OD_600_ of samples from cultures grown exponentially at OD < 0.2 for at least three generations. *Bcen* culture doubling times were observed to be less reproducible than those of *E*.*coli*, and showed day-to-day variation in all media, regardless of pre-culture history, number of generations in exponential phase or presence of antibiotics. Doubling times (minutes ± standard deviation) of Nel13-based strains were: SOC—60 ± 5 (37°), LB—108 ± 8 (30°), 75 ± 4 (37°); MGCC—110 ± 13 (30°), 76 ± 5 (37°); MglyC—144 ± 9 (30°), 91 ± 13 (37°). Doubling times of J2315-based strains were 3–4 minutes longer in all media.

The colony-appearance assay consisted of spreading cultured or transformed cells on solid LB or SOB medium, incubating them at 37°C and counting the colonies two or three times per day; the time at which colony number reached its maximum was taken as the colony-appearance time for purposes of comparison. Viability was estimated by applying cells from dilutions of SOB cultures to LB agar in two ways—by spreading using glass beads, and as a 10μl drop—followed by incubation at 37°C; colony counts per OD unit were calculated.

### DAPI staining

Samples of 10μl taken from exponential SOB cultures at OD_600_ ~0.2 were applied to polylysine-coated slides and allowed to dry at room temperature. After three rinses in M9 salts and drying in air, the cells were fixed with a drop of methanol and allowed to dry, then covered with 5μl 2μg/ml DAPI and a cover slip and viewed by phase-contrast and fluorescence microscopy using a DAPI broad filter.

### Marking origin regions for visualization

In most experiments, MGCC medium was inoculated from freshly-grown colonies at a concentration which ensured cells were still growing exponentially following overnight incubation at 30°C. Cells from the overnight cultures were diluted to OD_600_ 0.05 in 25ml MGCC and incubated to OD_600_ ~0.10. ParB::FP production was then induced by addition of arabinose. Arabinose concentrations and induction periods appropriate for optimum signal:background ratio were determined empirically, according to whether ParB::FP was used for origin marking, whether one ParB::FP was being produced or two simultaneously, and whether normal or disrupted Par function prevailed in the cells observed. Induction was usually arrested by addition of further glucose or by resuspension in MGCC, but occasional omission of this step proved not to be detrimental. Cells in which the c3 Par system was disrupted (Δ*parAB*c3 and extra *parS*c3) grew erratically in MGCC but reproducibly in SOB; the latter medium was used in this case.

Microscopy Culture samples were centrifuged and the cells resuspended in ~1/30 volume of medium. 1–2 μl was then applied to a 1% agarose-M9 salts layer on a microscope slide, spread by application of a coverslip and viewed under oil-immersion by phase contrast and epi-fluorescence microscopy. Microscopy was carried out in two laboratories: that of Dr J. Errington (Centre for Bacterial cell Biology, Newcastle-upon-Tyne; Fig 3) and that of the authors' institute (Figs 3–6). At the former, cells were observed with a Zeiss Axiovert M200 microscope equipped with a 300W Xenon lamp and a Zeiss Plan-Neofluar 100x/1.30 objective. The filters used were: Chroma 49002 ET-EGFP (exciter ET470/40X, dochroic T495LP, emitter ET525/50M) and 49008 ET-mCherry (ET560/40X; T585LP; ET630/75M), Schott UV GG385 and Schott IR KG5. Images were captured with a Photometrics Coolsnap HQ monochrome camera and analyzed using MetaMorph V.6.2r6. At the latter, cells were observed using a 100x oil-immersion objective (Plan Achromat, 1.4 NA; Olympus) the equivalent was an Olympus X81 wide-field inverted microscope equipped with an Olympus phase-contrast 100x/1.4 objective. The light source was a monochromator (Polychrome V; Till Photonics GmbH) with a 150W Xenon lamp used with a 15nm bandwidth. For two-colour experiments, multiband dichroic mirrors (Chroma BGR 69002) were used, and specific single-band emission filters (GFP 520/40, mCherry 632/60) were mounted on a motorized wheel. Images were captured with a Roper Coolsnap 2 camera and processed using Metamorph and ImageJ software.

Upon deposition on slides, *Bcen* cells tend to amass to form large groups in which cell dimensions cannot be accurately determined; accordingly we included only isolated cells or those in small groups in analyses of focus position. Cell poles were located by contrast difference in the grey-scale and the line connecting them was drawn using the ImageJ Straight function. Cells with septal constrictions were considered to be unitary unless septation was estimated to be more than two-thirds complete or the nascent cells were not aligned. Focus positions were determined as local fluorescence maxima at least two-fold higher than background using the plot-profile function of ImageJ. Distances were obtained from pixel size (64.5 nm). The initial pole-focus distance was determined by perpendicular projection to the line (Straight) from the focus maximum closest to a pole; second focus position was measured relative to the same pole.

### Determination of base-pair frequency gradients

Cultures of Nel13and its Δ*parA1*c1 derivative FBP7 were harvested after three generations of balanced exponential growth (72 and 136 minute doubling times respectively) to OD_600_ ~0.2 and, for Nel13, after 8 hours of incubation in stationary phase. DNA was purified as described and subjected to high-throughput sequencing by the Imagif Platform (Gif-sur-Yvette) using Illumina technology. Base-pair frequencies from the read density profiles were binned (10kb—c1 and c2, 1kb—c3) to generate base-pair gradients for the three chromosomes.

## Supporting Information

S1 Table Iterons of c2, c3 and p1. Iteron-like repeat sequences were located using the Operations/Analyze Molecule function of Clone Manager 9. < indicates reverse orientation of the iteron relative to the others. Only those repeats found near the origin are shown. Others are found throughout the genome, including a second c2 iteron cluster 140kb anticlockwise from the origin one shown here.
(DOCX)

S2 Table Cell cycle parameters. **A**—DNA per cell Cultures of *Bcen* J2315 that had grown exponentially for 4–5 generations to the optical densities shown were sampled for assay of DNA (3, 6 and 9 ml for J2315 and N13; 3.7 and 7.4 ml for *E*.*coli*; taken into ice-cold tubes with NaN3, final concentration 10mM) and of cell number (50 μL to 450 μL filtered M9 salts). Parallel cultures of *E*. *coli* K12 C600 in M9 glucose (0.4%) supplemented with thiamin, leucine and threonine were used as a standard of known cell cycle parameters to validate the assays. Cell concentration was determined, after further 100-fold dilution, using a Partec CyFlow cytometer; *Bcen* samples were assayed immediately since these cells tend to lyse during even short storage periods. DNA was assayed chemically using the Burton diphenylamine reaction [64], essentially as described by Bipatnath et al [28] with minor modifications [65], with salmon sperm DNA as a standard. S.e. values are standard errors of the mean of the DNA assays. The genome / cell value obtained for *E*. *coli* is close to the value of 1.9 reported for this species growing at 1 generation / hr [33]. **B**—Initiation age **C**—Segregation age.
(DOCX)

S3 Table Construction of *par* gene deletions. The *parAB*c2 deletion was made by strand-overlap extension PCR to create a fragment comprising the first 23 codons of *parA* fused to the last 17 codons of *parB* with ~1kb of the natural sequence flanking either end. The fragment was inserted into suicide vector pEX18Tc and introduced by triparental mating into *Bcen* J2315. Exconjugants selected for single-crossover plasmid integration by Tet^R^ were streaked on drug-free medium and screened for second-crossover plasmid excision by Tet^S^; one of three Tet^S^ isolates proved by PCR to have retained the *parAB* deletion. The *parAB*c3 and *parA*Bp1deletions were made by successive insertion of the upstream and downstream flanks of each *parAB* operon on either side of the cat gene in pCM351-cat. Nel13 was transformed to Cam^R^ with the respective plasmids (pDAG820 and 819), and transformants were screened for TetS then tested for retention of the deletions by PCR as above.
(DOCX)

S1 Fig Map of the *ori*c1 region. Relevant portions of the to-scale map above are expanded below. DnaA boxes were assigned on the basis of similarity to the *E*. *coli* consensus, justified by the identity of the residues determining DnaA-box recognition (S3 Fig). DnaA-boxes are shown as pennants: shaded—TTATCCACA, numbered—numbers correspond to positions of alteration to the canonical DnaA box. AT-rich regions are present in all four origin regions (see S2 Fig); although their significance is unknown their presence within a very GC-rich genome strongly suggests a role of duplex melting in replication control. 7-, 9-, and 10-mers are clustered sequence repeats (≤ 1 mismatch) of unknown significance in the *ori* region—CTGTGCA, ATCCGCGCW, CATGCGGCCG respectively; the 7-mers appear clustered also in the c2 and c3 origin regions, suggesting a regulatory function common to the three chromosomes. The exact location of the origin is not clear. For example, a second DnaA box cluster near nt1 and the GC-skew minimum might indicate the true *ori* better than that predicted by Ori-finder (Fig 1C).
(DOCX)

S2 Fig Maps of the *ori*c2, -c3 and—p1 regions, designed as in S1 Fig Blue triangles on both to-scale and expanded maps are 7mers like those in c1. Red triangles are iterons of consensus sequence (bold, 100% conserved; capital, ≥ 90%, small, < 90%): c2 –ctCCCGAAAAacCTCACCTtt, c3 –tCCCATAAacggntACCtnt, p1 –tgTCGTtCYTCCAGCGAtg See S1 Table for details. GC min* denotes the minimum disparity predicted by Ori-finder and indicates that it differs slightly from the GC skew minimum (obtained using http://gcat.davidson.edu/DGPB/gc_skew/gc_skew.html). As with c1, the exact location of the c2 origin might correspond to the iteron region rather than that 4kb away predicted by Ori-finder (*ori*- c2*).
(DOCX)

S3 Fig Alignment of the DNA-interaction domains of FtsK and DnaA. DnaA: yellow highlights show signature DnaA family protein residues; residues concluded from mutagenesis and DNA-binding studies [66] to specify site-specific binding are shown in red. FtsK: yellow highlights show residues conserved in Gram-negative bacteria; grey highlights show residues invariant in Gram-negative and positive bacteria; residues indicated from FtsK/KOPS co-crystals and from mutagenesis data to specify site-specific contacts with KOPS are shown in red and blue respectively [25].
(DOCX)

S4 Fig Relative copy numbers of c1, c2, c3 and p1. DNA was purified [10] from cells of J2315 growing exponentially at OD_600_ ~0.1 in LB. The DNA was cleaved to completion with *Apa*LI and the fragments in 0.5 μg and 1 μg of digested DNA (left and right lanes) were resolved by agarose gel electrophoresis (0.8%, TBE buffer), transferred to a nitrocellulose membrane (Qbiogen), fixed by UV irradiation, pre-incubated in 0.5M NaHPO_4_ pH 7.2-1mM EDTA-7% SDS-1% BSA with 0.1mg/ml sonicated and denatured calf-thymus DNA for 2h at 65°C, and hybridized with a mixture of ^32^P-labelled probes representing the origin and terminus of each replicon. Probe DNAs obtained as gel-purified PCR fragments were labelled with ^32^P by random primer extension (NEBlot kit, New England Biolabs) and added to the pre-hybridization mixture. After 12–14 h at 65°C the membrane was washed in 2% SSPE-1% SDS at 65°C for 30 min, again at room temperature, then twice in 0.2% SSPE-0.1% SDS. After phosphorimaging, the bands were quantified and their intensities corrected for probe length and specific activitiy. Bands corresponding to the origins and termini are indicated on the left and right respectively. The Table shows relative numbers of physically distinct replicons in an average cell, as determined from ratios of terminus probes hybridized. The plasmid to chromosome origin ratios were 0.72 (p1/c1), 0.79 (p1/c2) and 1.15 (p1/c3). The sequence of the p1 *ter* probe was taken from a site opposite the origin, although it is unknown whether p1 replication is uni- or bi-directional; because p1 is small any error owing to replication being unidirectional is negligible.
(DOCX)

S5 Fig Partition function of fluorescent ParB derivatives. Dubarry et al [10] determined the partition activity of *Bcen* ParB proteins by measuring the rates of loss of unstable mini-F plasmids carrying *Bcen parS* sites from dividing *E*. *coli* cells (strain DH10B) that express *Bcen parA* and *parB* genes from a second plasmid. The *parB*::*gfp*/*chfp* fusions used here to visualize *Bcen ori* regions were substituted for the native *parB* genes in these plasmids, and tested for partition activity in parallel with the original *parB*+ derivatives, using the same conditions. The *parAB*c1 and *parAB*c2 genes are carried by the multicopy vector, pBBR1mcs5, and the *parAB*c3 genes by the moderate copy number vector, pAM238. **A**—c1 *parA*B (pDAG562; black circles) and *parAB*::*chfp* (pDAG583; red) with mini-F pDAG551 (single *parS*c1 site). **B**—c2 *parAB* (pDAG563; grey) and *parAB*::*egfp* (pDAG584; green) with pDAG555 (four *parS*c2 sites); c2 *parAB*-g8c (pDAG566; black) and *parAB*-g8c::*chfp* (pDAG587; red) with pDAG552 (single *parS* site): g8c is a silent mutation in the *parS* site internal to the *parB* gene, which is presumed to raise *parB* expression above wild type. **C**—c3 *parAB* (pDAG560; black) and *parAB*c3::*egfp* (pDAG585; green) with pDAG553 (single *parS* site): this ParB/*parS* system was replaced by that of phage P1 in the experiments reported here. The loss rates were measured twice, with the bars showing the spread of values. The dotted line shows spontaneous loss of the mini-F vector (pDAG203) with no *parS*.
(DOCX)

S6 Fig Significance of c1 and c2 focus distribution differences. The 1- and 2-focus data of Figs 3B and 4A were sorted into cell length classes of 0.1 and 0.2 μm respectively, giving the distributions shown below. Visual inspection shows a consistent tendency for the leading tail of the distributions to contain a higher fraction of cells showing c1 foci than that of cells showing c2 foci, and for the trailing tail to contain a higher fraction of cells with c2 foci than that of cells with c1 foci. This observation suggests that single c1 foci do not persist as long into the cell cycle as single c2 foci and that double c1 foci appear sooner in the cycle than double c2 foci, i.e., that on average c1 origins segregate before c2 origins. However, the central portion of the distributions, containing the bulk of the data, did not show clear differences between c1 and c2. Application of the Student t-test (two-tailed), in Excel, to the entire distributions showed that for the data of Fig 3B, obtained from separately cultured cells, the mean size of c1 1-focus cells, 1.98 μm, exceeded that of c2 1-focus cells, 1.93 μm, at the limit of the 95% confidence level (p = 0.04); the mean size of c1 2-focus cells, 2.65 μm, was lower than that of c2 2-focus cells, 2.75 μm (p = 0.006). For the data of Fig 4A, obtained for c1 and c2 in the same cells, the same test did not show differences between the means that were significant at the 95% confidence level—p = 0.16 and 0.14 for 1-focus and 2-focus cells respectively: this resulted from the coincidence of focus number for both c1 and c2 in the large majority of cells. Application of the χ2 test to assess the significance of differences apparent in parts of the distributions yielded the following results: **A**—cells with c2 > 2.3 μm 49, < 2.3 μm 262 cf. expected from c1 distribution, 34.2, 276.8 respectively—χ2 7.19 cf. 3.84 null value at 95% confidence; proportion single c2 focus cells > 2.3 μm higher than proportion single c1 focus cells **B**—cells with c1 > 2.3 μm 338, < 2.3 μm 124 cf. expected from c2 distribution, 368.35 and 93.65 - χ2 12.34 cf. 3.84; proportion double c1 focus cells < 2.3 μm higher than proportion double c2 focus cells **C**—cells with c2 > 1.9 μm 35, < 1.9 μm 78 cf. expected from c1 distribution, 26.3, 86.7 - χ2 3.75 cf. 3.84; proportion single c2 focus cells > 1.9 μm not significantly higher than proportion single c1 focus cells **D**—cells with c1 > 2.0 μm 336, < 2.0 μm 94 cf. expected from c2 distribution 357.2, 71.8 - χ2 8.21 cf. 3.84; proportion double c1 focus cells < 2.0 μm higher than proportion double c2 focus cells.
(DOCX)

S7 Fig Western blot assay of ParB protein abundance. Extracts of cells from exponentially-growing LB cultures of the *Bcen* strains shown were fractionated by SDS-PAGE and the proteins analysed by standard Western blotting using polyclonal antibodies raised against specific ParB peptides (by Eurogentec). For each antibody the ParB band and a cross-reacting host protein band are indicated by black and white arrowheads respectively. Band intensities were within the linear range of applied protein concentration. Their relative intensities were normalized to the cross-reacting band, and these ratios plotted relative to that of the Nel13 wt. ΔAc1, ΔABc2 and ΔABp1 denote strains deleted for these par genes; pMMB-pSc denote Nel13 transformed with the pMMBΔ vector and its derivatives carrying the indicated single *parS* sites or clusters (marked +).
(DOCX)

S8 Fig Cell abnormalities characteristic of the Δ*parA*c1 cells. **A**–Phase contrast image of a typical Δ*parA*c1 triplet: one daughter cell has elongated without septation while the second cell has suspended septation, leaving two rounded, contracted cells. **B**–Time course of long cell rupture: 200 minutes after deposit on agar-medium, a bleb appears in the long cell, announcing imminent explosion with loss of wall integrity and cell contents, seen at 208 mins. **C**—DAPI staining: the four first images show Δ*parA*c1 triplets with the nucleoid uncompacted and clear in the elongated cell, while dense in one rounded cell and totally or partly absent in the second; the fifth image shows wild-type cells.
(DOCX)
