# Supplementary material for: Orderly Replication and Segregation of the Four Replicons of Burkholderia cenocepacia J2315
Source: PLoS Genet. 2016 Jul 18;12(7):e1006172. doi: 10.1371/journal.pgen.1006172 (PMC4948915; doi:10.1371/journal.pgen.1006172)
Supplement: S1 Table — Iteron-like repeat sequences were located using the Operations/Analyze Molecule function of Clone Manager 9. < indicates reverse orientation of the iteron relative to the others. Only those repeats found near the origin are shown. Others are found throughout the genome, including a second c2 iteron cluster 140kb anticlockwise from the origin one shown here. (DOCX) [file pgen.1006172.s001.docx]

**Table S1** Iterons of c2, c3 and p1

**c2 origin cluster c3 origin cluster p1 origin cluster**

nt.

5864 CTCCCGAAAACGCTCACCGGT 151 CCCCATATCCGTCTACCTTT 92376 TGACGTTCTTCCAGCGATG

6219 GTCCCGAAAATACTCACCTCA 330 TCCCATAACCTGCTACCTTC 92465 TGTCGTTCTTCCAGCGATG

6307 CTCCTGAAAACGCTCACCTTA 356 TCCCATAGGAGGGTACCTGT 92496 GGTCGTTCCTCCAGCGATG

6365 CTCCCGAAAAACCTCACCGTA 422 GCCCATAAACGGCAACCATT 92541 TGTCGTTCTTCCAGCGATG

6462 CCCCCGAAAAACCTCACCTTT 448 CCCCATAAAAGGTAACCTGT 46 TATCGTTCTTCCAGCGAGC<

6518 CCCCCGAAAAATCTCACCTGT 493 TCCCATAGACAGG-ACCGGT < 138 CCTCGTTCCTCCAGCGTGA<

6614 GTCCCGAAAAGCCTCACCTTT 545 TCCCATAACCGGCTACCTTC 189 TATCGTGCCTCCAGCGATG<

6658 CTCCCGGAAAATCTCACCTGT 571 CCCCATAACCGGCTACCCAA 231 TCTCGTCTCTCCAGCGATG<

6705 GCCCCGAAAAAGCTCACCTTA 635 GCCCATAAAAGGCTACCTTC

6757 CTCCCGAAAAACCTCACCTTT 661 TCCCATAGACGACTACCTAA

6862 GTCCCGTGAAACCTCACCTTT 699 TCCCATAACCGGATACGTTC

6928 GTCCTGCAAATTCTCACCTAT 720 CCCCATATCCACAACCCTGA

7006 GCCCCGAAAACCCTCACCTTT 808 TCCCATAAATGGGTACCGAA

7138 CTCCCGTAAAACTTCACCTTG 939 CCCCATAAACCGCTACCAAT

1113 TCCCATAAGTTTCTACGTTT <

1154 TCCCATAAACGGGAACCTCT <

**consensus** ct**CC**C**G**aA**AA**ac**CTCACC**Ttt t**CCCATA**aacggctA**C**Ctnt tgT**CGT**tCY**TCCAGCG**Atg

capital bold - 100%

capital - ≥ 85%

small - 61-85%

small grey - 50-60%

Iteron-like repeat sequences were located using the Operations/Analyze Molecule function of Clone Manager 9. < indicates reverse orientation of the iteron relative to the others. Only those repeats found near the origin are shown. Others are found throughout the genome, including a second c2 iteron cluster 140kb anticlockwise from the origin one shown here.
