# Supplementary material for: Orderly Replication and Segregation of the Four Replicons of Burkholderia cenocepacia J2315
Source: PLoS Genet. 2016 Jul 18;12(7):e1006172. doi: 10.1371/journal.pgen.1006172 (PMC4948915; doi:10.1371/journal.pgen.1006172)
Supplement: S2 Table — A—DNA per cell Cultures of Bcen J2315 that had grown exponentially for 4–5 generations to the optical densities shown were sampled for assay of DNA (3, 6 and 9 ml for J2315 and N13; 3.7 and 7.4 ml for E.coli; taken into ice-cold tubes with NaN3, final concentration 10mM) and of cell number (50 μL to 450 μL filtered M9 salts). Parallel cultures of E. coli K12 C600 in M9 glucose (0.4%) supplemented with thiamin, leucine and threonine were used as a standard of known cell cycle parameters to validate the assays. Cell concentration was determined, after further 100-fold dilution, using a Partec CyFlow cytometer; Bcen samples were assayed immediately since these cells tend to lyse during even short storage periods. DNA was assayed chemically using the Burton diphenylamine reaction [64], essentially as described by Bipatnath et al [28] with minor modifications [65], with salmon sperm DNA as a standard. S.e. values are standard errors of the mean of the DNA assays. The genome / cell value obtained for E. coli is close to the value of 1.9 reported for this species growing at 1 generation / hr [33]. B—Initiation age C—Segregation age. (DOCX) [file pgen.1006172.s002.docx]

**Table S2** Cell cycle parameters

A - DNA per cell

_______________________________________________________________________

strain medium doubling OD_600_ cells/ml µg DNA genome / cell

time (mins) x 10^8^ /ml culture (G_C_) ± s.e.

_______________________________________________________________________

J2315 LB 75 0.201 2.16 3.04 1.61 ± 0.17

Nel13 MGCC 74 0.289 3.75 4.93 1.50 ± 0.16

*E. coli* K12 M9-glu 63 0.224 2.11 2.10 1.98 ± 0.18

(C600)

_______________________________________________________________________

Cultures of *Bcen* J2315 that had grown exponentially for 4-5 generations to the optical densities shown were sampled for assay of DNA (3, 6 and 9 ml for J2315 and N13; 3.7 and 7.4 ml for *E.coli*; taken into ice-cold tubes with NaN_3_, final concentration 10mM) and of cell number (50 µL to 450 µL filtered M9 salts). Parallel cultures of *E. coli* K12 C600 in M9 glucose (0.4%) supplemented with thiamin, leucine and threonine were used as a standard of known cell cycle parameters to validate the assays. Cell concentration was determined, after further 100-fold dilution, using a Partec CyFlow ^®^ cytometer; *Bcen* samples were assayed immediately since these cells tend to lyse during even short storage periods. DNA was assayed chemically using the Burton diphenylamine reaction ([1](#_ENREF_1)), essentially as described by Bipatnath *et al* ([2](#_ENREF_2)) with minor modifications ([3](#_ENREF_3)), with salmon sperm DNA as a standard. S.e. values are standard errors of the mean of the DNA assays. The genome / cell value obtained for *E. coli* is close to the value of 1.9 reported for this species growing at 1 generation / hr ([4](#_ENREF_4)).

B - Initiation age To calculate the age at initiation of the major replicons, c1 and c2, we assume first that replication of both is initiated at effectively the same time, and second, that the *Bcen* genome may be treated as a single large chromosome replicated a at a proportionately higher speed, i.e. in the time normally taken to replicate c1, with the later-replicating c3 compensating to some degree for the smaller size of c2. This enables us to calculate cell cycle parameters from doubling time, replication time and DNA mass per cell in the same way that they have been for monochromosomal *E. coli*.

From Cooper and Helmstetter ([4](#_ENREF_4)), cell age (0 for newborn cells, 1 for cells in the last stage of division) at initiation of chromosome replication in exponentially-growing cells is given by: a_i_ = 1 + n - (C+D)/τ where C is time taken to replicate the chromosome, D is the interval between termination of replication and division, τ is cell doubling time, and n is the next integer lower than (C+D)/τ; D is obtained from G_C_ = (τ/C.ln2)(2^(C+D)/τ^ -2^D/τ^) as:

D = τ.{log[(G_C_.C.ln2) / τ.(2^C/τ^ − 1)] /log2}, where G_C_ is the average number of genome equivalents per cell, obtained as in A, above.

The G_C_ values in Table S2A were used to calculate D and a_i_ , whence :

D a_i_

J2315 in LB 20 mins 0.93 (~5 mins before division)

Nel13 in MGCC 12 mins 0.03 (~2 mins after division)

We consider these values as indicative only.

C - Segregation age For exponentially dividing cells, the cell age at duplication of any entity can be calculated from the fractions of cells containing one and two copies of that entity, provided the fractions carrying 0 or >2 copies are negligible, as in the present case, using age = 1 - (lnF/ln2) where F = 1 + (2-focus cells / total cells).

replicon experiment 1-focus 2-foci F age

R = Chfp G = Gfp

c1 73 429 1.855 0.11

c1-R + c2-G

c2 113 389 1.775 0.17

0.16

c2 91 364 1.800 0.15

c2-R + c3-G

c3 270 185 1.407 0.51

0.48

c3 263 242 1.479 0.44

c3-G + p1-R

p1 186 319 1.632 0.29

These values confirm the segregation order derived by inspection of the data of Fig. 4.
