# Supplementary material for: Orderly Replication and Segregation of the Four Replicons of Burkholderia cenocepacia J2315
Source: PLoS Genet. 2016 Jul 18;12(7):e1006172. doi: 10.1371/journal.pgen.1006172 (PMC4948915; doi:10.1371/journal.pgen.1006172)
Supplement: S3 Table — The parABc2 deletion was made by strand-overlap extension PCR to create a fragment comprising the first 23 codons of parA fused to the last 17 codons of parB with ~1kb of the natural sequence flanking either end. The fragment was inserted into suicide vector pEX18Tc and introduced by triparental mating into Bcen J2315. Exconjugants selected for single-crossover plasmid integration by TetR were streaked on drug-free medium and screened for second-crossover plasmid excision by TetS; one of three TetS isolates proved by PCR to have retained the parAB deletion. The parABc3 and parABp1deletions were made by successive insertion of the upstream and downstream flanks of each parAB operon on either side of the cat gene in pCM351-cat. Nel13 was transformed to CamR with the respective plasmids (pDAG820 and 819), and transformants were screened for TetS then tested for retention of the deletions by PCR as above. (DOCX) [file pgen.1006172.s003.docx]

**Table S3** Construction of *par* gene deletions

| *par* locus  deleted | vector | nt co-ordinates:  flank 1 flank 2 | | *parA(B)*  replaced by: | plasmid  (suicide in *Bcen*) | screen for deletion | *Δpar* strain |
| --- | --- | --- | --- | --- | --- | --- | --- |
| **parA*c1 | *pCM351-cat | 28389-29673 | 30479-31764 | cat | pRF91 | Tet^S^ | FBP47 |
| *parAB*c2 | pEX18Tc | 1540-2608 | 3101-4140 | - | pEX-  ΔparABK2 | Tet^S^ | Nel11 |
| *parAB*c3 | pCM351-cat | 3186-4685 | 875407-1391 | cat | pDAG820 | Tet^S^ | Nel42 |
| *parAB*p1 | pCM351-cat | 91342-92645 | 1652-2983 | cat | pDAG819 | Tet^S^ | Nel43 |

* Described by Dubarry et al ([5](#_ENREF_5)).

The *parAB*c2 deletion was made by strand-overlap extension PCR to create a fragment comprising the first 23 codons of *parA* fused to the last 17 codons of *parB* with ~1kb of the natural sequence flanking either end. The fragment was inserted into suicide vector pEX18Tc and introduced by triparental mating into Bcen J2315. Exconjugants selected for single-crossover plasmid integration by Tet^R^ were streaked on drug-free medium and screened for second-crossover plasmid excision by Tet^S^; one of three Tet^S^ isolates proved by PCR to have retained the *parAB* deletion.

The *parAB*c3 and *parAB*p1deletions were made by successive insertion of the upstream and downstream flanks of each *parAB* operon on either side of the *cat* gene in pCM351-cat. Nel13 was transformed to Cam^R^ with the respective plasmids (pDAG820 and 819), and transformants were screened for Tet^S^ then tested for retention of the deletions by PCR as above.
