# Supplementary material for: Orderly Replication and Segregation of the Four Replicons of Burkholderia cenocepacia J2315
Source: PLoS Genet. 2016 Jul 18;12(7):e1006172. doi: 10.1371/journal.pgen.1006172 (PMC4948915; doi:10.1371/journal.pgen.1006172)
Supplement: S1 Fig — Relevant portions of the to-scale map above are expanded below. DnaA boxes were assigned on the basis of similarity to the E. coli consensus, justified by the identity of the residues determining DnaA-box recognition (S3 Fig). DnaA-boxes are shown as pennants: shaded—TTATCCACA, numbered—numbers correspond to positions of alteration to the canonical DnaA box. AT-rich regions are present in all four origin regions (see S2 Fig); although their significance is unknown their presence within a very GC-rich genome strongly suggests a role of duplex melting in replication control. 7-, 9-, and 10-mers are clustered sequence repeats (≤ 1 mismatch) of unknown significance in the ori region—CTGTGCA, ATCCGCGCW, CATGCGGCCG respectively; the 7-mers appear clustered also in the c2 and c3 origin regions, suggesting a regulatory function common to the three chromosomes. The exact location of the origin is not clear. For example, a second DnaA box cluster near nt1 and the GC-skew minimum might indicate the true ori better than that predicted by Ori-finder (Fig 1C). (DOCX) [file pgen.1006172.s004.docx]

**Fig. S1** Map of the *ori*c1 region.


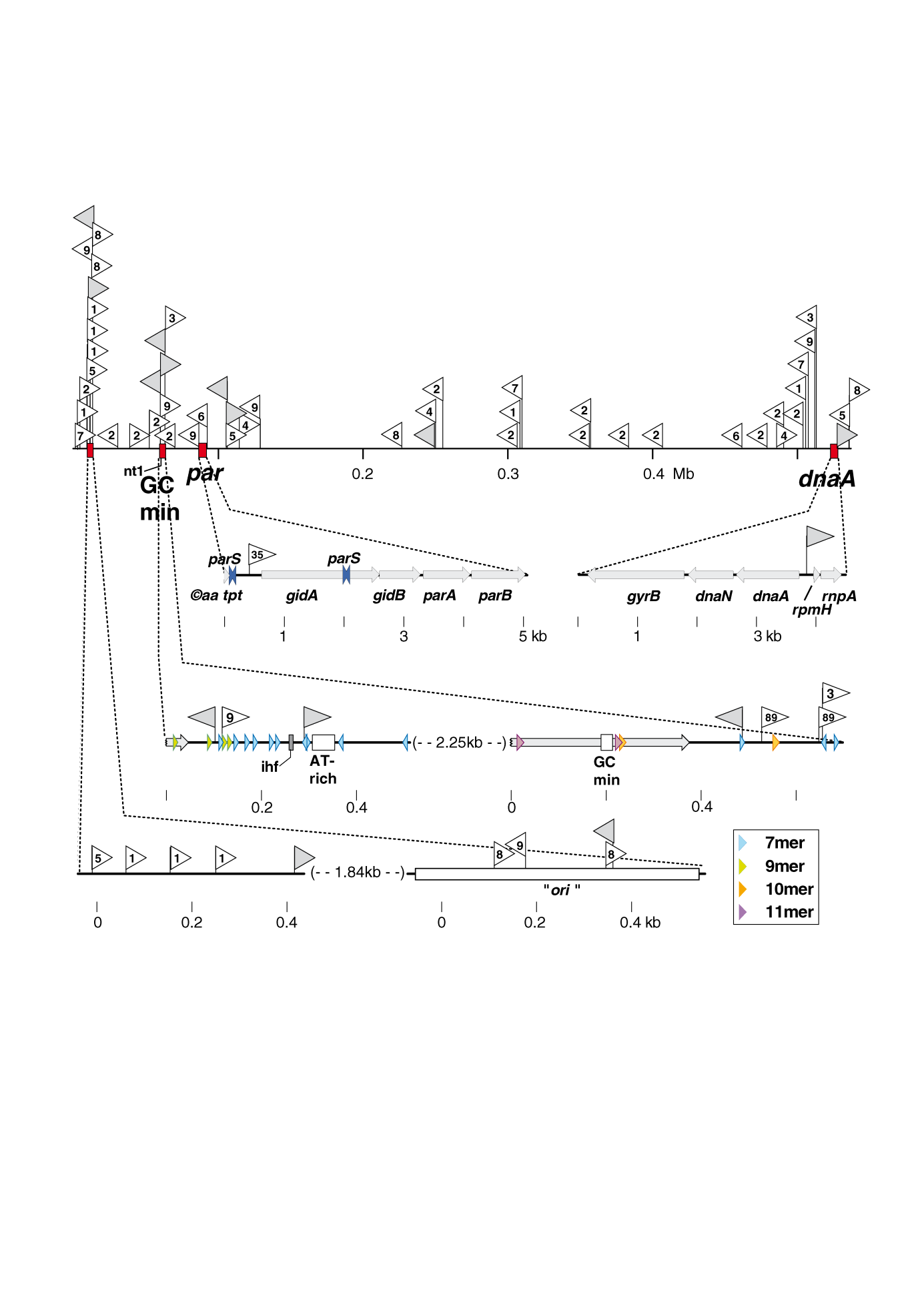


Relevant portions of the to-scale map above are expanded below. DnaA boxes were assigned on the basis of similarity to the *E. coli* consensus, justified by the identity of the residues determining DnaA-box recognition (Fig. S3). DnaA-boxes are shown as pennants: shaded – TTATCCACA, numbered – numbers correspond to positions of alteration to the canonical DnaA box. AT-rich regions are present in all four origin regions (see Fig S2); although their significance is unknown their presence within a very GC-rich genome strongly suggests a role of duplex melting in replication control. 7-, 9-, and 10-mers are clustered sequence repeats (≤ 1 mismatch) of unknown significance in the *ori* region – CTGTGCA, ATCCGCGCW, CATGCGGCCG respectively; the 7-mers appear clustered also in the c2 and c3 origin regions, suggesting a regulatory function common to the three chromosomes. The exact location of the origin is not clear. For example, a second DnaA box cluster near nt1 and the GC-skew minimum might indicate the true *ori* better than that predicted by Ori-finder (Fig 1C).
