# Supplementary material for: Orderly Replication and Segregation of the Four Replicons of Burkholderia cenocepacia J2315
Source: PLoS Genet. 2016 Jul 18;12(7):e1006172. doi: 10.1371/journal.pgen.1006172 (PMC4948915; doi:10.1371/journal.pgen.1006172)
Supplement: S2 Fig — Red triangles are iterons of consensus sequence (bold, 100% conserved; capital, ≥ 90%, small, < 90%): c2 –ctCCCGAAAAacCTCACCTtt, c3 –tCCCATAAacggntACCtnt, p1 –tgTCGTtCYTCCAGCGAtg See S1 Table for details. GC min* denotes the minimum disparity predicted by Ori-finder and indicates that it differs slightly from the GC skew minimum (obtained using http://gcat.davidson.edu/DGPB/gc_skew/gc_skew.html). As with c1, the exact location of the c2 origin might correspond to the iteron region rather than that 4kb away predicted by Ori-finder (ori- c2*). (DOCX) [file pgen.1006172.s005.docx]

**Fig. S2** Maps of the *ori*c2, -c3 and - p1 regions, designed as in Fig. S1.


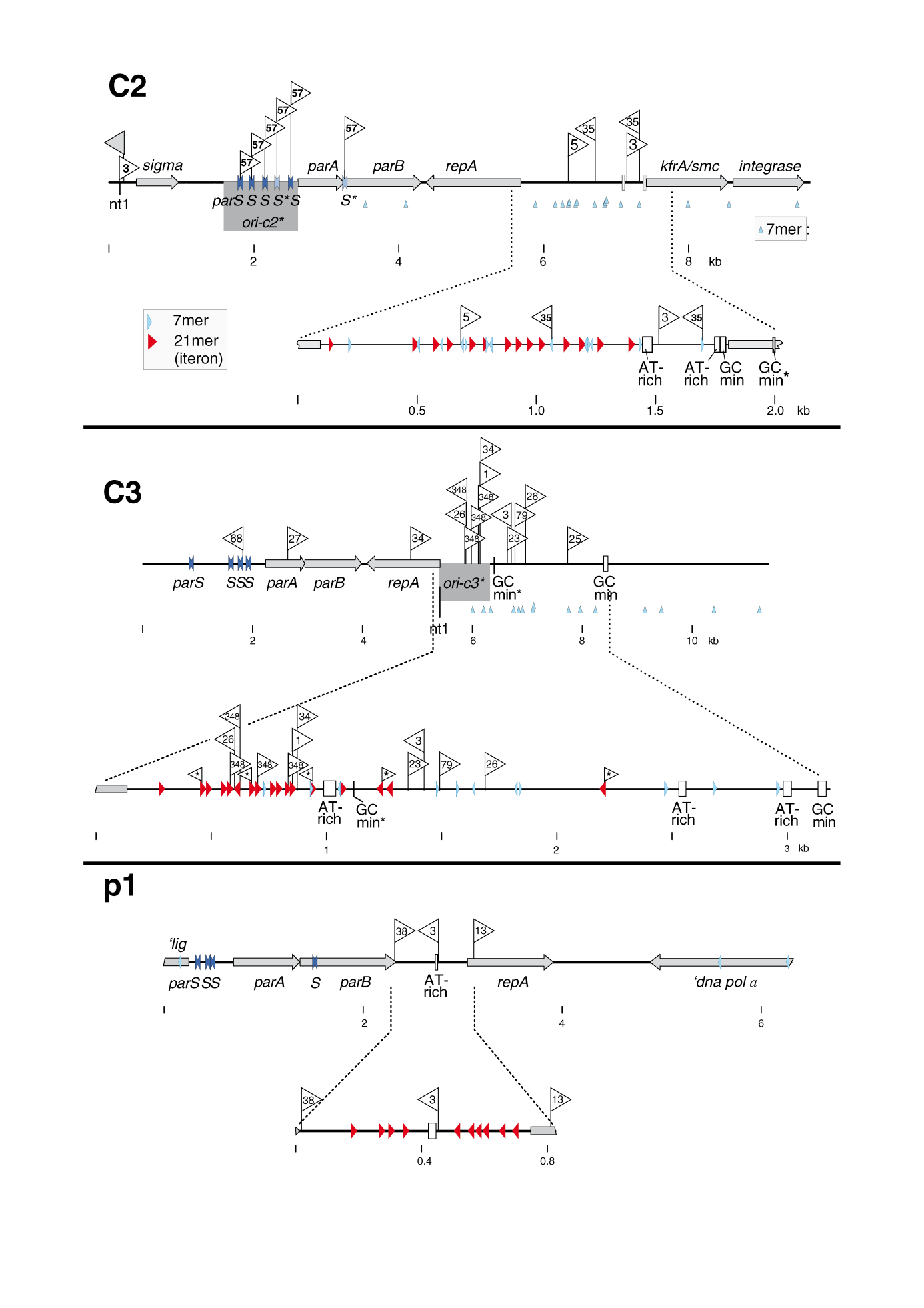


Blue triangles on both to-scale and expanded maps are 7mers like those in c1. Red triangles are iterons of consensus sequence (bold, 100% conserved; capital, ≥ 90%, small, < 90%):

c2 – ct**CC**C**G**AA**AA**ac**CTCACC**Ttt, c3 – t**CCCATA**AacggntA**C**Ctnt,

p1 – tgT**CGT**tCY**TCCAGCG**Atg

See Table S1 for details.

GC min* denotes the minimum disparity predicted by Ori-finder and indicates that it differs slightly from the GC skew minimum (obtained using <http://gcat.davidson.edu/DGPB/gc_skew/gc_skew.html>). As with c1, the exact location of the c2 origin might correspond to the iteron region rather than that 4kb away predicted by Ori-finder (*ori-c2**).
