# Supplementary material for: Orderly Replication and Segregation of the Four Replicons of Burkholderia cenocepacia J2315
Source: PLoS Genet. 2016 Jul 18;12(7):e1006172. doi: 10.1371/journal.pgen.1006172 (PMC4948915; doi:10.1371/journal.pgen.1006172)
Supplement: S3 Fig — FtsK: yellow highlights show residues conserved in Gram-negative bacteria; grey highlights show residues invariant in Gram-negative and positive bacteria; residues indicated from FtsK/KOPS co-crystals and from mutagenesis data to specify site-specific contacts with KOPS are shown in red and blue respectively [25]. (DOCX) [file pgen.1006172.s006.docx]

**Fig. S3** Alignment of the DNA-interaction domains of FtsK and DnaA

.

**DnaA *E.coli* 421 SLPEIGDAFGGRDHTTVLHACRKIEQLREESHDIKEDFSNLIRTL**

**DnaA *Bcen* 479 SLPEIGELFGGRDHTTVLHAVRKIADERSKDAQLNHELHVLEQTL**

**FtsK *E.coli* 1266 DPLFDQAVQFVTEKRKASISGVQRQFRIGYNRAARIIEQMEAQGIVSEQGHNGNREVLAPPPFD**

**FtsK *Bcen* 705 DPLYDQAVEIVIKNRRASISLVQRHLRIGYNRAARLLEQMEQSGLVSAMSSSGNREILVPARDAE**

DnaA: yellow highlights show signature DnaA family protein residues; residues concluded from mutagenesis and DNA-binding studies ([6](#_ENREF_6)) to specify site-specific binding are shown in red.

FtsK: yellow highlights show residues conserved in Gram-negative bacteria; grey highlights show residues invariant in Gram-negative and positive bacteria; residues indicated from FtsK/KOPS co-crystals and from mutagenesis data to specify site-specific contacts with KOPS are shown in red and blue respectively ([7](#_ENREF_7)).
