# Supplementary material for: Orderly Replication and Segregation of the Four Replicons of Burkholderia cenocepacia J2315
Source: PLoS Genet. 2016 Jul 18;12(7):e1006172. doi: 10.1371/journal.pgen.1006172 (PMC4948915; doi:10.1371/journal.pgen.1006172)
Supplement: S4 Fig — DNA was purified [10] from cells of J2315 growing exponentially at OD600 ~0.1 in LB. The DNA was cleaved to completion with ApaLI and the fragments in 0.5 μg and 1 μg of digested DNA (left and right lanes) were resolved by agarose gel electrophoresis (0.8%, TBE buffer), transferred to a nitrocellulose membrane (Qbiogen), fixed by UV irradiation, pre-incubated in 0.5M NaHPO4 pH 7.2-1mM EDTA-7% SDS-1% BSA with 0.1mg/ml sonicated and denatured calf-thymus DNA for 2h at 65°C, and hybridized with a mixture of 32P-labelled probes representing the origin and terminus of each replicon. Probe DNAs obtained as gel-purified PCR fragments were labelled with 32P by random primer extension (NEBlot kit, New England Biolabs) and added to the pre-hybridization mixture. After 12–14 h at 65°C the membrane was washed in 2% SSPE-1% SDS at 65°C for 30 min, again at room temperature, then twice in 0.2% SSPE-0.1% SDS. After phosphorimaging, the bands were quantified and their intensities corrected for probe length and specific activitiy. Bands corresponding to the origins and termini are indicated on the left and right respectively. The Table shows relative numbers of physically distinct replicons in an average cell, as determined from ratios of terminus probes hybridized. The plasmid to chromosome origin ratios were 0.72 (p1/c1), 0.79 (p1/c2) and 1.15 (p1/c3). The sequence of the p1 ter probe was taken from a site opposite the origin, although it is unknown whether p1 replication is uni- or bi-directional; because p1 is small any error owing to replication being unidirectional is negligible. (DOCX) [file pgen.1006172.s007.docx]

**Fig. S4** Relative copy numbers of c1, c2, c3 and p1.


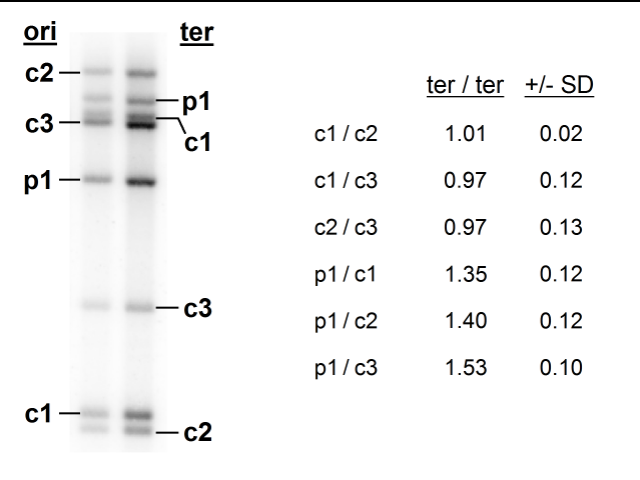


DNA was purified ([8](#_ENREF_8)) from cells of J2315 growing exponentially at OD_600_ ~0.1 in LB. . The DNA was cleaved to completion with *Apa*LI and the fragments in 0.5 µg and 1 µg of digested DNA (left and right lanes) were resolved by agarose gel electrophoresis (0.8%, TBE buffer), transferred to a nitrocellulose membrane (Qbiogen), fixed by UV irradiation, pre-incubated in 0.5M NaHPO4 pH 7.2-1mM EDTA-7% SDS-1% BSA with 0.1mg/ml sonicated and denatured calf-thymus DNA for 2h at 65°C, and hybridized with a mixture of ^32^P-labelled probes representing the origin and terminus of each replicon. Probe DNAs obtained as gel-purified PCR fragments were labelled with ^32^P by random primer extension (NEBlot kit, New England Biolabs) and added to the pre-hybridization mixture. After 12-14 h at 65°C the membrane was washed in 2% SSPE-1% SDS at 65°C for 30 min, again at room temperature, then twice in 0.2% SSPE-0.1% SDS. After phosphorimaging, the bands were quantified and their intensities corrected for probe length and specific activitiy. Bands corresponding to the origins and termini are indicated on the left and right respectively. The Table shows relative numbers of physically distinct replicons in an average cell, as determined from ratios of terminus probes hybridized. The plasmid to chromosome origin ratios were 0.72 (p1/c1), 0.79 (p1/c2) and 1.15 (p1/c3). The sequence of the p1 ter probe was taken from a site opposite the origin, although it is unknown whether p1 replication is uni- or bi-directional; because p1 is small any error owing to replication being unidirectional is negligible.
