# Supplementary material for: Orderly Replication and Segregation of the Four Replicons of Burkholderia cenocepacia J2315
Source: PLoS Genet. 2016 Jul 18;12(7):e1006172. doi: 10.1371/journal.pgen.1006172 (PMC4948915; doi:10.1371/journal.pgen.1006172)
Supplement: S5 Fig — Dubarry et al [10] determined the partition activity of Bcen ParB proteins by measuring the rates of loss of unstable mini-F plasmids carrying Bcen parS sites from dividing E. coli cells (strain DH10B) that express Bcen parA and parB genes from a second plasmid. The parB::gfp/chfp fusions used here to visualize Bcen ori regions were substituted for the native parB genes in these plasmids, and tested for partition activity in parallel with the original parB+ derivatives, using the same conditions. The parABc1 and parABc2 genes are carried by the multicopy vector, pBBR1mcs5, and the parABc3 genes by the moderate copy number vector, pAM238. A—c1 parAB (pDAG562; black circles) and parAB::chfp (pDAG583; red) with mini-F pDAG551 (single parSc1 site). B—c2 parAB (pDAG563; grey) and parAB::egfp (pDAG584; green) with pDAG555 (four parSc2 sites); c2 parAB-g8c (pDAG566; black) and parAB-g8c::chfp (pDAG587; red) with pDAG552 (single parS site): g8c is a silent mutation in the parS site internal to the parB gene, which is presumed to raise parB expression above wild type. C—c3 parAB (pDAG560; black) and parABc3::egfp (pDAG585; green) with pDAG553 (single parS site): this ParB/parS system was replaced by that of phage P1 in the experiments reported here. The loss rates were measured twice, with the bars showing the spread of values. The dotted line shows spontaneous loss of the mini-F vector (pDAG203) with no parS. (DOCX) [file pgen.1006172.s008.docx]

**Fig. S5**  Partition function of fluorescent ParB derivatives.


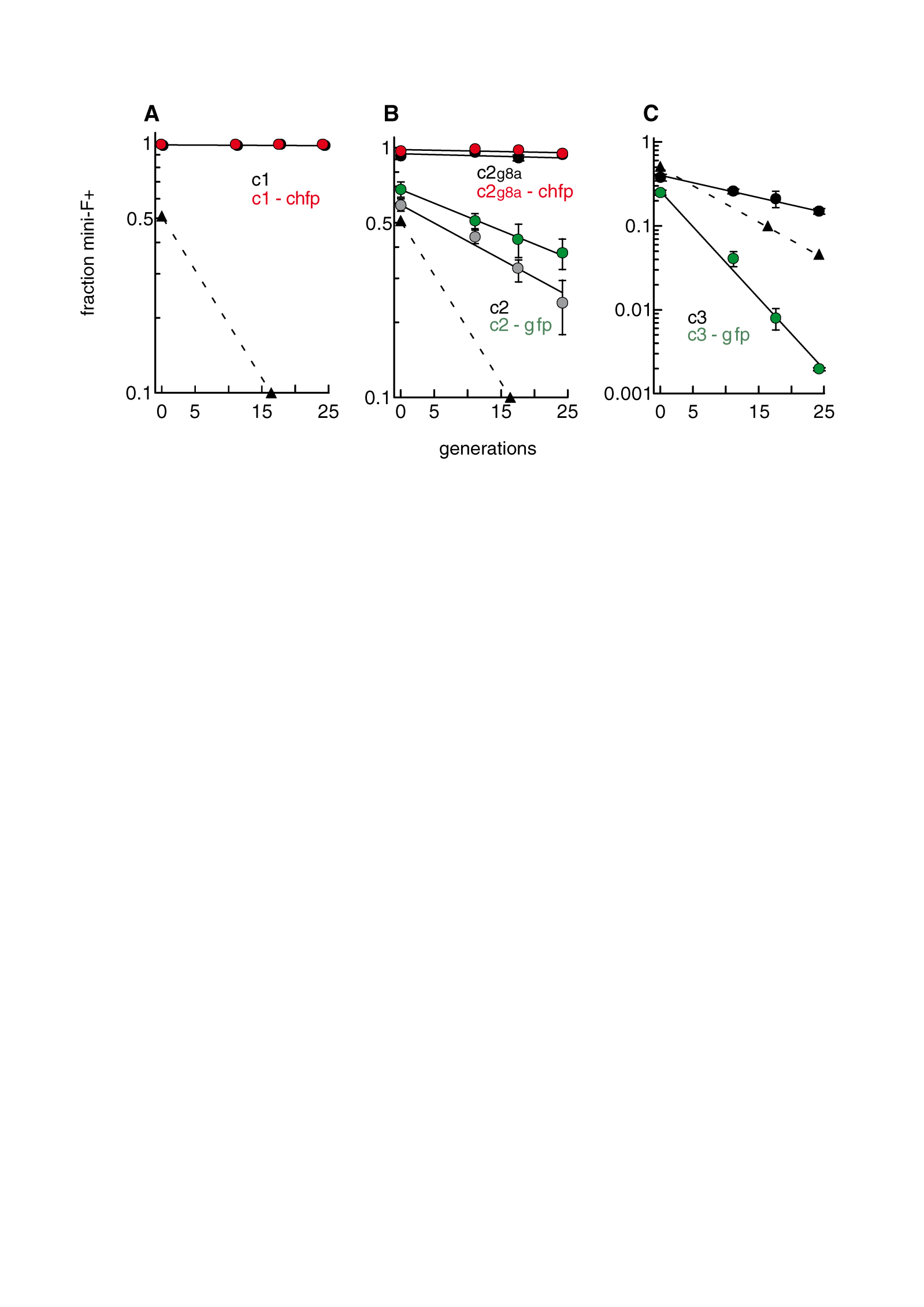


Dubarry et al ([8](#_ENREF_8)) determined the partition activity of *Bcen* ParB proteins by measuring the rates of loss of unstable mini-F plasmids carrying *Bcen* *parS* sites from dividing *E. coli* cells (strain DH10B) that express *Bcen* *parA* and *parB* genes from a second plasmid. The *parB*::*gfp*/*chfp* fusions used here to visualize *Bcen* *ori* regions were substituted for the native *parB* genes in these plasmids, and tested for partition activity in parallel with the original *parB^+^* derivatives, using the same conditions. The *parAB*c1 and parABc2 genes are carried by the multicopy vector, pBBR1mcs5, and the *parAB*c3 genes by the moderate copy number vector, pAM238. **A -** c1 *parAB* (pDAG562; black circles) and *parAB*::*chfp* (pDAG583; red) with mini-F pDAG551 (single *parS*c1 site). **B -** c2 *parAB* (pDAG563; grey) and *parAB*::*egfp* (pDAG584; green) with pDAG555 (four *parS*c2 sites); c2 *parAB*-g8c (pDAG566; black) and *parAB*-g8c::chfp (pDAG587; red) with pDAG552 (single *parS* site): g8c is a silent mutation in the *parS* site internal to the *parB* gene, which is presumed to raise *parB* expression above wild type. **C -** c3 *parAB* (pDAG560; black) and *parAB*c3::*egfp* (pDAG585; green) with pDAG553 (single *parS* site): this ParB/*parS* system was replaced by that of phage P1 in the experiments reported here. The loss rates were measured twice, with the bars showing the spread of values. The dotted line shows spontaneous loss of the mini-F vector (pDAG203) with no *parS*.
