# Supplementary material for: Orderly Replication and Segregation of the Four Replicons of Burkholderia cenocepacia J2315
Source: PLoS Genet. 2016 Jul 18;12(7):e1006172. doi: 10.1371/journal.pgen.1006172 (PMC4948915; doi:10.1371/journal.pgen.1006172)
Supplement: S6 Fig — The 1- and 2-focus data of Figs 3B and 4A were sorted into cell length classes of 0.1 and 0.2 μm respectively, giving the distributions shown below. Visual inspection shows a consistent tendency for the leading tail of the distributions to contain a higher fraction of cells showing c1 foci than that of cells showing c2 foci, and for the trailing tail to contain a higher fraction of cells with c2 foci than that of cells with c1 foci. This observation suggests that single c1 foci do not persist as long into the cell cycle as single c2 foci and that double c1 foci appear sooner in the cycle than double c2 foci, i.e., that on average c1 origins segregate before c2 origins. However, the central portion of the distributions, containing the bulk of the data, did not show clear differences between c1 and c2. Application of the Student t-test (two-tailed), in Excel, to the entire distributions showed that for the data of Fig 3B, obtained from separately cultured cells, the mean size of c1 1-focus cells, 1.98 μm, exceeded that of c2 1-focus cells, 1.93 μm, at the limit of the 95% confidence level (p = 0.04); the mean size of c1 2-focus cells, 2.65 μm, was lower than that of c2 2-focus cells, 2.75 μm (p = 0.006). For the data of Fig 4A, obtained for c1 and c2 in the same cells, the same test did not show differences between the means that were significant at the 95% confidence level—p = 0.16 and 0.14 for 1-focus and 2-focus cells respectively: this resulted from the coincidence of focus number for both c1 and c2 in the large majority of cells. Application of the χ2 test to assess the significance of differences apparent in parts of the distributions yielded the following results: A—cells with c2 > 2.3 μm 49, < 2.3 μm 262 cf. expected from c1 distribution, 34.2, 276.8 respectively—χ2 7.19 cf. 3.84 null value at 95% confidence; proportion single c2 focus cells > 2.3 μm higher than proportion single c1 focus cells B—cells with c1 > 2.3 μm 338, < 2.3 μm 124 cf. expected from c2 di [file pgen.1006172.s009.docx]

**Fig. S6** Significance of c1 and c2 focus distribution differences

A

B

C

D

The 1- and 2-focus data of Figures 3B and 4A were sorted into cell length classes of 0.1 and 0.2 µm respectively, giving the distributions shown below. Visual inspection shows a consistent tendency for the leading tail of the distributions to contain a higher fraction of cells showing c1 foci than that of cells showing c2 foci, and for the trailing tail to contain a higher fraction of cells with c2 foci than that of cells with c1 foci. This observation suggests that single c1 foci do not persist as long into the cell cycle as single c2 foci and that double c1 foci appear sooner in the cycle than double c2 foci, i.e., that on average c1 origins segregate before c2 origins.

However, the central portion of the distributions, containing the bulk of the data, did not show clear differences between c1 and c2. Application of the Student t-test (two-tailed), in Excel, to the entire distributions showed that for the data of Fig 3B, obtained from separately cultured cells, the mean size of c1 1-focus cells, 1.98 µm, exceeded that of c2 1-focus cells, 1.93 µm, at the limit of the 95% confidence level (p = 0.04); the mean size of c1 2-focus cells, 2.65 µm, was lower than that of c2 2-focus cells, 2.75 µm (p = 0.006).

For the data of Fig 4A, obtained for c1 and c2 in the same cells, the same test did not show differences between the means that were significant at the 95% confidence level - p = 0.16 and 0.14 for 1-focus and 2-focus cells respectively: this resulted from the coincidence of focus number for both c1 and c2 in the large majority of cells.

Application of the χ2 test to assess the significance of differences apparent in parts of the distributions yielded the following results:

A - cells with c2 > 2.3 µm 49, < 2.3 µm 262 cf. expected from c1 distribution, 34.2, 276.8 respectively -

χ2 7.19 cf. 3.84 null value at 95% confidence;

proportion single c2 focus cells > 2.3 µm higher than proportion single c1 focus cells

B - cells with c1 > 2.3 µm 338, < 2.3 µm 124 cf. expected from c2 distribution, 368.35 and 93.65 -

χ2 12.34 cf. 3.84;

proportion double c1 focus cells < 2.3 µm higher than proportion double c2 focus cells

C - cells with c2 > 1.9 µm 35, < 1.9 µm 78 cf. expected from c1 distribution, 26.3, 86.7 -

χ2 3.75 cf. 3.84;

proportion single c2 focus cells > 1.9 µm not significantly higher than proportion single c1 focus cells

D - cells with c1 > 2.0 µm 336, < 2.0 µm 94 cf. expected from c2 distribution 357.2, 71.8 -

χ2 8.21 cf. 3.84;

proportion double c1 focus cells < 2.0 µm higher than proportion double c2 focus cells.
