# Supplementary material for: Orderly Replication and Segregation of the Four Replicons of Burkholderia cenocepacia J2315
Source: PLoS Genet. 2016 Jul 18;12(7):e1006172. doi: 10.1371/journal.pgen.1006172 (PMC4948915; doi:10.1371/journal.pgen.1006172)
Supplement: S7 Fig — Extracts of cells from exponentially-growing LB cultures of the Bcen strains shown were fractionated by SDS-PAGE and the proteins analysed by standard Western blotting using polyclonal antibodies raised against specific ParB peptides (by Eurogentec). For each antibody the ParB band and a cross-reacting host protein band are indicated by black and white arrowheads respectively. Band intensities were within the linear range of applied protein concentration. Their relative intensities were normalized to the cross-reacting band, and these ratios plotted relative to that of the Nel13 wt. ΔAc1, ΔABc2 and ΔABp1 denote strains deleted for these par genes; pMMB-pSc denote Nel13 transformed with the pMMBΔ vector and its derivatives carrying the indicated single parS sites or clusters (marked +). (DOCX) [file pgen.1006172.s010.docx]

**Fig. S7** Western blot assay of ParB protein abundance.


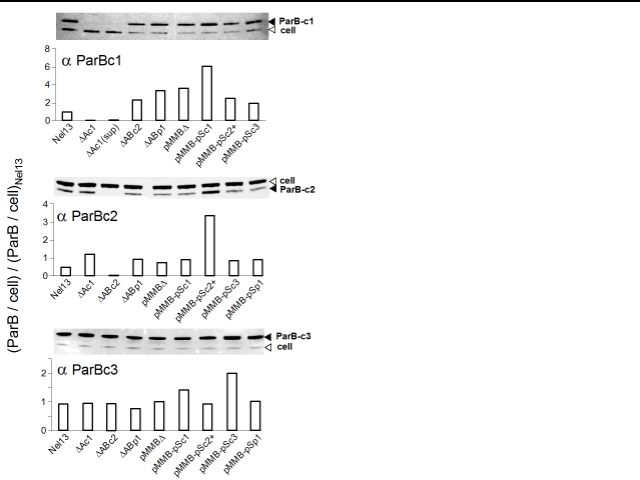


Extracts of cells from exponentially-growing LB cultures of the *Bcen* strains shown were fractionated by SDS-PAGE and the proteins analysed by standard Western blotting using polyclonal antibodies raised against specific ParB peptides (by Eurogentec). For each antibody the ParB band and a cross-reacting host protein band are indicated by black and white arrowheads respectively. Band intensities were within the linear range of applied protein concentration. Their relative intensities were normalized to the cross-reacting band, and these ratios plotted relative to that of the Nel13 wt. ΔAc1, ΔABc2 and ΔABp1 denote strains deleted for these *par* genes; pMMB-pSc denote Nel13 transformed with the pMMBΔ vector and its derivatives carrying the indicated single *parS* sites or clusters (marked +).
