# Supplementary material for: Orderly Replication and Segregation of the Four Replicons of Burkholderia cenocepacia J2315
Source: PLoS Genet. 2016 Jul 18;12(7):e1006172. doi: 10.1371/journal.pgen.1006172 (PMC4948915; doi:10.1371/journal.pgen.1006172)
Supplement: S8 Fig — A–Phase contrast image of a typical ΔparAc1 triplet: one daughter cell has elongated without septation while the second cell has suspended septation, leaving two rounded, contracted cells. B–Time course of long cell rupture: 200 minutes after deposit on agar-medium, a bleb appears in the long cell, announcing imminent explosion with loss of wall integrity and cell contents, seen at 208 mins. C—DAPI staining: the four first images show ΔparAc1 triplets with the nucleoid uncompacted and clear in the elongated cell, while dense in one rounded cell and totally or partly absent in the second; the fifth image shows wild-type cells. (DOCX) [file pgen.1006172.s011.docx]

**Fig. S8** Cell abnormalities characteristic of *ΔparA*c1 cells.


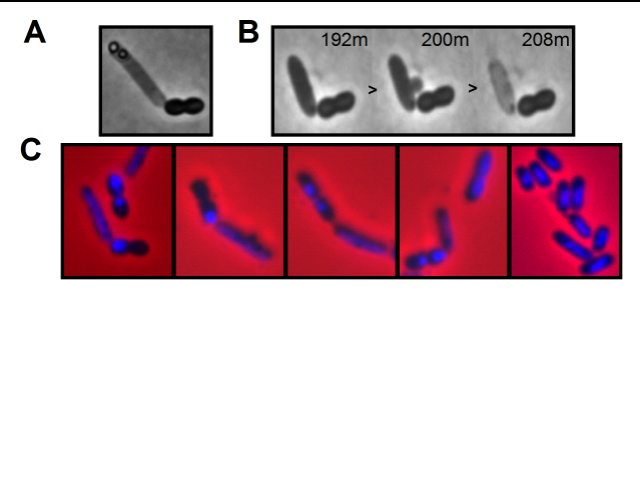


A – Phase contrast image of a typical Δ*parA*c1 triplet: one daughter cell has elongated without septation while the second cell has suspended septation, leaving two rounded, contracted cells. B – Time course of long cell rupture: 200 minutes after deposit on agar-medium, a bleb appears in the long cell, announcing imminent explosion with loss of wall integrity and cell contents, seen at 208 mins. C - DAPI staining: the four first images show Δ*parA*c1 triplets with the nucleoid uncompacted and clear in the elongated cell, while dense in one rounded cell and totally or partly absent in the second; . the fifth image shows wild-type cells.
